# What empirical research has been undertaken on the ethics of clinical research in India? A systematic scoping review and narrative synthesis

**DOI:** 10.1136/bmjgh-2020-004729

**Published:** 2021-05-18

**Authors:** Sangeetha Paramasivan, Philippa Davies, Alison Richards, Julia Wade, Leila Rooshenas, Nicola Mills, Alba Realpe, Jeffrey Pradeep Raj, Supriya Subramani, Jonathan Ives, Richard Huxtable, Jane M Blazeby, Jenny L Donovan

**Affiliations:** 1 Population Health Sciences, Bristol Medical School, University of Bristol, Bristol, UK; 2 University Hospitals Bristol NHS Foundation Trust, NIHR ARC West, Bristol, UK; 3 Medical Research Council (MRC) ConDuCT-II Trials Methodology Hub, Bristol Medical School, University of Bristol, Bristol, UK; 4 Department of Clinical Pharmacology, Seth GS Medical College and KEM Hospital, Mumbai, Maharashtra, India; 5 Institute of Biomedical Ethics and History of Medicine, University of Zurich, Zurich, Switzerland; 6 Centre for Ethics in Medicine, University of Bristol, Bristol, UK; 7 University Hospitals Bristol NHS Foundation Trust, NIHR Bristol Biomedical Research Centre, Bristol, UK

**Keywords:** systematic review

## Abstract

**Introduction:**

The post-2005 rise in clinical trials and clinical research conducted in India was accompanied by frequent reports of unethical practices, leading to a series of regulatory changes. We conducted a systematic scoping review to obtain an overview of empirical research pertaining to the ethics of clinical trials/research in India.

**Methods:**

Our search strategy combined terms related to ethics/bioethics, informed consent, clinical trials/research and India, across nine databases, up to November 2019. Peer-reviewed research exploring ethical aspects of clinical trials/research in India with any stakeholder groups was included. We developed an evidence map, undertook a narrative synthesis and identified research gaps. A consultation exercise with stakeholders in India helped contextualise the review and identify additional research priorities.

**Results:**

Titles/Abstracts of 9699 articles were screened, full text of 282 obtained and 80 were included. Research on the ethics of clinical trials/research covered a wide range of topics, often conducted with little to no funding. Studies predominantly examined what lay (patients/public) and professional participants (eg, healthcare staff/students/faculty) know about topics such as research ethics or understand from the information given to obtain their consent for research participation. Easily accessible groups, namely ethics committee members and healthcare students were frequently researched. Research gaps included developing a better understanding of the recruitment-informed consent process, including the doctor-patient interaction, in multiple contexts and exploring issues of equity and justice in clinical trials/research.

**Conclusion:**

The review demonstrates that while a wide range of topics have been studied in India, the focus is largely on assessing knowledge levels across different population groups. This is a useful starting point, but fundamental questions remain unanswered about informed consent processes and broader issues of inequity that pervade the clinical trials/research landscape. A priority-setting exercise and appropriate funding mechanisms to support researchers in India would help improve the clinical trials/research ecosystem.

**Key questions**

**What is already known?**

- The increase in the number of clinical trials and clinical research conducted in India after 2005 was accompanied by many reports of ethical misconduct, with bioethics reports and health activism prompting a series of regulatory changes by the government.
- While there was a corresponding increase in empirical research on various ethical aspects of clinical trials/research in India, little was known about the scope of this research or what areas of research required further attention to improve the clinical trials/research ecosystem.

**What are the new findings?**

- Research on ethical aspects of clinical trials/research in India was often carried out with limited to no funding, covered a wide range of topics but with a focus on knowledge assessments of lay and professional groups on topics such as research ethics, and leaned on easily accessible groups such as ethics committee members and healthcare students for study populations.
- A range of research gaps were identified, facilitated by a consultation exercise with key stakeholders from India, and included developing a better understanding of the different components of the recruitment and informed consent process, such as the doctor-patient interaction, developing models of informed consent specific to the Indian context and exploring issues such as equity and justice within the context of clinical trials/research.

**What do the new findings imply?**

- There is a need to move from knowledge assessments towards addressing other fundamental questions about recruitment, informed consent, equity and justice.
- The large number of research gaps identified warrants a locally led priority-setting exercise as well as appropriate funding mechanisms to support researchers in India to undertake clinical trials/research methodology and ethics-related research.

## Introduction

International clinical trials recruit participants from low-income and middle-income countries (LMICs) for economic, pragmatic and scientific reasons.^1^ Post-2005, when the World Trade Organisation-Trade Related Intellectual Property Rights agreement became fully binding for India, the number of clinical trials approved by the Indian government’s regulatory authority, Central Drugs Standard Control Organisation, began to increase,^2^ peaking in 2010 followed by a sharp decline to 2013^3^ (online supplemental file 1). An identical pattern of growth and contraction was observed in India’s clinical trial sector’s growth rate, in research using clinicaltrials.gov data.^4^

10.1136/bmjgh-2020-004729.supp1 Supplementary data

The downward trend is attributed to the chain of events that began with unacceptable ethical practices, such as failure to obtain participants’ informed consent for trial participation,^5^ being reported nationally and internationally.^6–11^ In 2013, the Supreme Court of India intervened and briefly halted approvals for new clinical trials^12^ in response to concerns for participant autonomy and safety, and public interest litigations from non-governmental organisations.^13 14^ New regulations were introduced in 2013 as amendments to Schedule Y of the Drugs and Cosmetics Rules 1945,^15^ mandating measures such as registration of ethics committees^16^ and audio-visual (AV) recordings of the informed consent discussion,^17 18^ the latter being a requirement that is unique to India (see Gogtay *et al*^18^ for an overview of regulatory changes/requirements in India from 2005 to 2016). Also specific to India is that the term ‘clinical trial’ is limited to the study of ‘new drugs’ only, with Biomedical and Health Research (BMHR) referring to all other basic, applied, operational and clinical research^19^ (in contrast to broader definitions of ‘clinical trial’, which include medical, surgical and behavioural interventional research).^20 21^ The most recent regulatory changes outlined in the New Drugs and Clinical Trial (NDCT) Rules of 2019^19 22^ bring non-drug-related research (ie, BMHR) within the regulatory ambit for the first time^19 23^ (previously, regulatory mechanisms in India were principally focused on ‘new drug’ research). The NDCT Rules^19^ also separate the ethics and governance processes for clinical trials and bioavailability/bioequivalence studies from those for BMHR studies. For instance, two different types of ethics committees, each with separate authorities responsible for their registration and monitoring, will approve the two groups of studies. It is also now mandatory for BMHR ethics committees and academic clinical trials to adhere to the Indian Council for Medical Research’s National Ethical Guidelines for Biomedical and Health Research Involving Human Participants.^24 25^

Given this backdrop, there is a large body of theoretical bioethics literature and commentary by researchers, advocacy groups and bioethicists, covering topics such as lessons learnt from conducting clinical trials,^26–28^ ‘standard care’ in clinical trials,^29 30^ structure of the clinical trial industry,^31^ informed consent placed within the wider socioeconomic context,^32^ role of ethics committees^33^ and ensuring appropriate compensation mechanisms.^34^ There has also been a corresponding increase in empirical research on the ethics of clinical trials specifically and clinical research more broadly (henceforth clinical trials/research) in India, which has not been comprehensively reviewed. We therefore sought to summarise this body of research evidence through a systematic scoping review and narrative synthesis to help identify research gaps.

## Methods

We undertook a systematic scoping review following the established six-step framework by Arksey and O’Malley,^35^ drawing from recommendations to enhance the methodology^36–38^ and adhering to the Preferred Reporting Items for Systematic Reviews and Meta-analysis extension for scoping reviews^39^ (online supplemental file 2).

10.1136/bmjgh-2020-004729.supp2 Supplementary data

An initial systematic review of clinical trial informed consent interventions in India (PROSPERO registration: CRD42017068966) was amended to a systematic ‘scoping’ review (not within PROSPERO’s remit, hence withdrawn) of research on the ethics of clinical trials/research in India, as the latter method is particularly useful when the aim is to map the evidence base in a broad but complex unreviewed area.^35 37 38^

### Identifying the research question

We sought to obtain an overview of the empirical evidence in relation to the ethics of conducting clinical trials/research in India. More specifically, we aimed:

- to map the empirical research undertaken on any ethical aspect of conducting clinical trials/research in India;
- to synthesise the key themes from this evidence base, with a focus on informed consent;
- to identify gaps to inform future research priorities.

### Identifying relevant studies

#### Inclusion criteria

The research questions were assessed in relation to the setting, population, phenomenon of interest and the study design of articles (online supplemental file 3). We included articles that reported (a) on original research in a peer-reviewed journal, (b) on India as a country for data collection (if study involved many countries, included if India-specific findings could be differentiated), (c) on ethical issues in relation to clinical trials/research and (d) with any key stakeholder groups—lay (public; clinical trials/research participants; patients/guardians), professional (healthcare/research faculty, students or practitioners; ethics committee members; regulatory/governmental agencies) or documents (informed consent forms; ethics applications).

10.1136/bmjgh-2020-004729.supp3 Supplementary data

#### Exclusion criteria

We excluded commentaries, ‘lessons learnt’ articles, abstracts, letters, audits (eg, Clinical Trials Registry-India audits,^40 41^ except when linked to an ethical issue), and studies from countries other than India (eg, studies exploring views of researchers from high-income countries undertaking research in LMICs).^42 43^ We excluded studies on the following topics:

- Willingness to participate (WTP) in clinical trials/research and recruitment-focussed studies, except when they considered ethical issues (there are other systematic reviews on WTP^44–46^; WTP components of included studies were not considered in this review).
- Informed consent/ethical issues in relation to procedures/treatment outside of clinical trials/research (eg, in routine surgery).^47 48^
- Pharmacovigilance (PV) studies (there are systematic reviews on PV^49^; PV components of included studies were not considered in this review).
- Other: studies on medical/healthcare/clinical ethics (ie, not in relation to clinical trials/research or research ethics) and research skills/capacity with professional groups (eg, healthcare students).^50 51^

No restrictions were applied based on language, age (children/adult), study design or quality of research.

#### Search strategy

We searched the following nine electronic bibliographic databases with no start date and up to 5 September 2017 and this was updated using the technique by Bramer and Bain^52^ to 12 November 2019: MEDLINE, Cochrane Library, Web of Science, Scopus, Embase, PsycINFO, Cumulative Index of Nursing and Allied Health Literature, International Bibliography of Social Sciences and Online Resource for Recruitment research in Clinical TriAls.^53^ Search terms relating to three domains were combined: (a) ethics, bioethics, informed consent; (b) clinical trials/research and (c) India. A comprehensive search strategy first developed on MEDLINE (SP) drew from systematic reviews on related topics,^54 55^ was refined by an information specialist (ARi) and adapted to the other databases (online supplemental file 4—MEDLINE search strategy). Searches included other South Asian countries to gather contextual information, but the review focused on India. We used a combination of Medical Subject Headings, text word searches and search strings using proximity indicators. We searched the reference lists of eligible research articles and ineligible key opinion/commentary pieces, and contacted authors of published conference abstracts to trace studies.

10.1136/bmjgh-2020-004729.supp4 Supplementary data

### Study selection

All articles identified from the databases and other sources were downloaded to EndNote-X9^56^ and duplicates removed. Following the original search in September 2017, one reviewer (SP) screened the titles and abstracts of all articles with a 20% random sample screened independently by a second reviewer (PD). There was a high level of agreement across the two reviewers (disagreement in 3 of 1292 articles), with discrepancies discussed and resolved. Full text of all relevant articles were obtained and screened independently by at least two authors (SP with NM, JW, LR). Discordance was again resolved through group discussion among all four reviewers. Where it was unclear if an article or a particular topic should be included (eg, biobanking, data sharing), a decision was made by meeting with two content experts (ethicists JI and RH) and reviewing the articles together. For the search and screening update in November 2019, SP carried out all steps.

### Charting the data: data extraction and quality assessment

A data extraction form was developed (SP) and independently applied by two reviewers (SP and ARe) on a sample of articles (n=10). The form was refined after discussion and captured the following information (SP, ARe, JPR, SS): authors, year of publication and data collection, location, study aim, topic area, population, study design/methods, participants and findings. Subsequently, further information was captured on (SP): (a) whether studies were conducted within the context of a real or hypothetical study/scenario and (b) whether they explored broad (eg, clinical trials/research, research ethics) or specific topics (eg, data sharing, compensation).

Two review authors (SP with LR, JW, PD, JPR, SS) independently assessed the quality^57^ of the majority of studies using the following tools: Critical Appraisal Skills Programme (CASP) checklist^58^ for qualitative studies; Appraisal tool for Cross-Sectional Studies (AXIS; adapted to have 14 items instead of 20)^59^ for quantitative studies and AXIS, CASP and a section of the Mixed Methods Appraisal Tool^60^ for mixed methods studies. Quality assessments were discussed to resolve discrepancies and used to summarise relevant methodological issues in the narrative synthesis.

### Collating, synthesising and reporting the results

We first quantified the data in relation to the study characteristics. Next, we created an evidence map to visualise the volume of studies by topic, population group and methods. Finally, we synthesised the quantitative and qualitative findings reported in included studies, using EndNote-X9^56^ for data management and MaxQDA-12^61^ for coding articles, and used narrative and thematic description to write detailed descriptive accounts. The synthesis broadly followed the categorisations in the evidence map, but looked across all included articles to provide a comprehensive account of research on a given topic.

### Consultation

The consultation phase, considered optional in scoping reviews,^35^ took place after the synthesis, with the aim of informing the review and ensuring local priorities and context were accounted for. We approached colleagues in India who were researchers, ethicists and representatives from advocacy groups, through prior networks or because they had authored seminal empirical and/or conceptual papers (online supplemental file 5—consultation members). Consultation was carried out via virtual conferencing, email and telephone. Findings and research gaps identified through the review were discussed. Key recommendations made by stakeholders were grouped by topic and incorporated in the manuscript, tables or supplements.

10.1136/bmjgh-2020-004729.supp5 Supplementary data

### Patient and public involvement

No patients or members of the public were involved in this review.

## Results

### Description of included studies

**Figure 1:**
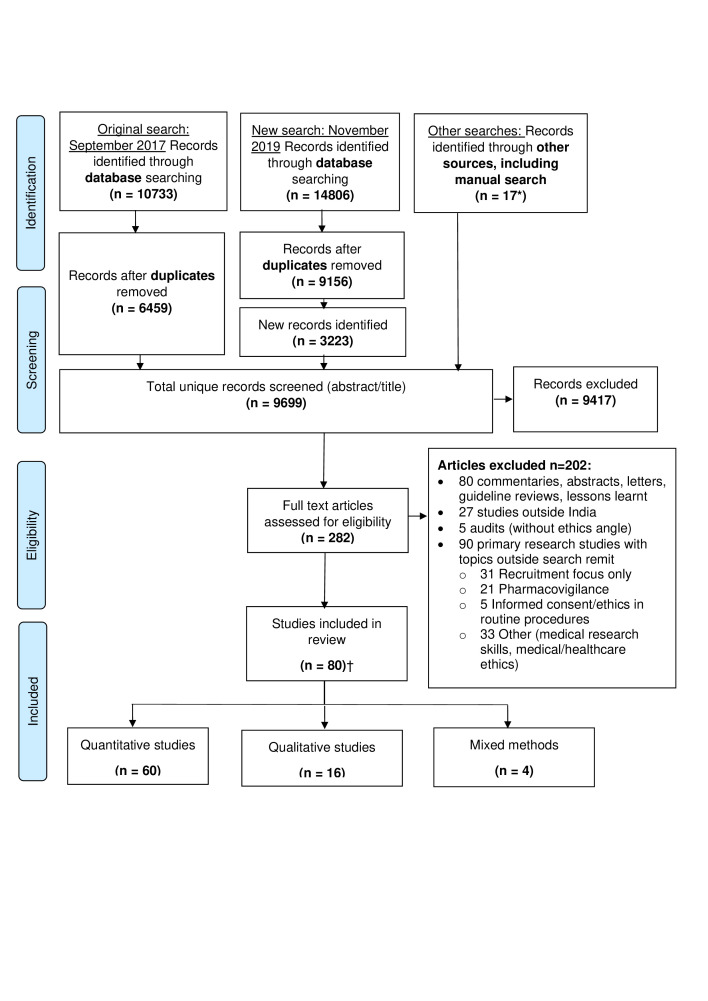
Preferred Reporting Items for Systematic Reviews and Meta-analysis flow diagram.^160^ *One study was identified through the consultation exercise. †This includes articles that reported on different aspects of the results derived from the same dataset^73 92 93 107 108^ or on different datasets obtained through the same grant.^113 114 120 126 127 160^

A total of 9699 unique records were identified (original, updated and manual searches), of which 282 full-text articles were assessed against the inclusion/exclusion criteria and 80 included^62–141^ (figure 1). Key study characteristics are summarised in table 1 (individual study details are in online supplemental file 6).

10.1136/bmjgh-2020-004729.supp6 Supplementary data

**Table 1:** Key characteristics of included studies

Key characteristics (total n=80)	N	%
**1. Location**		
***a. Type***		
Urban	**47**	**58.8**
Rural	**3**	**3.8**
Mixed	**3**	**3.8**
Not available*/Not applicable†	**27**	**33.8**
***b. Region***		
West	**24**	**30**
South	**21**	**26.3**
North	**10**	**12.5**
East	**2**	**2.5**
Mixed (two studies in west and south; two in west, south and north)	**4**	**5**
Pan India‡	**12**	**15**
Not available	**7**	**8.8**
**2. Methods**		
***a. Quantitative***	**60**	**75**
Surveys (inferential)	21	
Surveys (descriptive)	15	
Documents (descriptive)	13	
Documents (inferential)	4	
Other (documents, data, observation, RCT, websites)	7	
***b. Qualitative***	**16**	**20**
Interviews	10	
Interviews and focus groups	3	
Interviews and observations	2	
Interviews, observations, focus groups	1	
***c. Mixed methods***	**4**	**5**
Survey (descriptive) and interviews	2	
Survey (descriptive) and focus groups	1	
Survey (inferential) and focus groups	1	
**3. Population**		
***a. Professional***	**34**	**42.5**
Ethics committee members	8	
Researchers (two with CT investigators; two with clinical research professionals; one with CRO staff)	5	
Healthcare students (five with medical students; one each with nursing and pharmacy students)	7	
Healthcare faculty (two with dental faculty; one with medical faculty)	3	
Healthcare students and faculty (two with dental students and faculty; one with medical students and faculty)	3	
Healthcare service providers (one with healthcare faculty)	3	
Mixed professional groups	5	
***b. Lay***	**17**	**21.3**
RCT/CT participants (including parents/guardians, healthy volunteers)	6	
Cohort study participants (including parents/guardians)	3	
General public (including those accessed from hospitals)	6	
Specific patient groups (HIV-positive patients; mental health service inpatients)	2	
***c. Documents***	**22**	**27.5**
***d. Mixed*** (combination of lay, professional, documents)	**7**	**8.8**
**4. Journal**		
a. Published in India	**49**	**61.3**
b. Published in a high-income country	**29**	**36.3**
c. Unknown/not clear	**2**	**2.5**

*When information is not reported.

†When data collected is documents.

‡Includes surveys, documents, journal articles, websites that were not specific to one region.

CRO, contract research organisation; CT, clinical trial; RCT, randomised controlled trial.

Most studies were conducted in urban settings (47/80), in the western (24/80) and southern (21/80) parts of India. Studies were mainly quantitative (60/80), questionnaire surveys (36/60), conducted with professional groups (34/80) and appeared in journals published in India (49/80), primarily the *Indian Journal of Medical Ethics*^142^ and *Perspectives in Clinical Research*^143^ (n=15 and 16, respectively).

There were no research studies published on the ethical issues around conducting clinical trials/research until 2008, with a large proportion published a few years before and after the landmark regulatory changes of 2013 (53/80 were published 2011–2016; online supplemental file 1). Many studies did not mention the year of data collection (27/80) and of those that did, only a few were carried out in/after 2013 (17/53).

Corresponding authors of most studies were based within academic institutions (69/80; 15 outside India and 54 within India), primarily within Departments of Pharmacology of various Indian institutions (24/54). Seth Gordhandas Sunderdas Medical College and King Edward Memorial Hospital, Mumbai had the most number of corresponding authors (12/54), followed by Christian Medical College, Vellore (5/54). Two-thirds of studies (53/80) did not provide information on their funding source (26/53) or stated they did not receive any funding (27/53); of the remaining, 21 were funded/supported by international grants, 4 by intramural grants and 2 by pharmaceutical companies. There was no statement on conflicts of interest in 28 studies.

### Evidence map: research on ethical aspects of conducting clinical trials/research in India

We developed an evidence map that charts the total articles included (n=80) by the main focus of the topics and population covered in the studies, alongside the methods used (table 2).

**Table 2:** Evidence map of the number of primary and secondary research articles by topic and population group (studies explored multiple areas and have been categorised by main topic area studied)

				Quantitative		
				Qualitative		
				Mixed methods		
Population *Topic*			Lay(a)	Professional(b)	Mixed(a and b)	Total
A. Primary research: Knowledge (or awareness/comprehension), attitudes (or perceptions), practice (or behaviour)*						
***Comprehension of the informed consent form and/or verbal information provision in:***						
Real	Randomised Controlled Trial		2^65 116^†			2
	Clinical Trial		3^62 78 82^			3
	Cohort Study		2^87 117^	1^104^		3
Hypothetical	Randomised Controlled Trial		1^80^			1
	Clinical Trial			1^91^		1
***Knowledge, Attitudes, Practices in relation to:***						
Broad topics:	Clinical Trials		2^122 134^	3^75 100 129^		7
			1^88^	1^112^		
	Clinical Research		2^69 113^	1^131^		3
	Clinical Research Ethics and/or Ethics Committees			5^77 81 98 135 137^		5
Specific topics within:	Clinical Trials	Compensation for clinical trial related injury			1^125^‡	1
	Clinical Research Ethics	Ethical guidelines		1^101^		1
		Informed consent		1^138^		2
				1^114^		
		Ethics committees’ composition and/or functioning (incl. ethical review)		3^97 102 130^§		3
**Subtotal**			**13**	**18**	**1**	**32**
***A. Primary research: Perceptions, experiences, practices/processes¶ in relation to:***						
Real	Randomised Controlled Trial	Feasibility of informed consent procedure			1^70^	1
		Patient participation in / content of informed consent discussion (examined through audio-visual recordings)	1^83^			1
	Cohort study	Patient participation in informed consent discussion	1^111^			1
	Clinical Trial	Audio-visual recording of informed consent process (description and views)			1^79^	1
	Clinical Research	Recruitment experience/process and informed consent in bioavailability/bioequivalence studies			1^140^	1
Hypothetical	Clinical Trial	Audio-visual recording of informed consent process (acceptability)	1^72^			1
	Clinical Trial and Biobanking Research (meaning of consent, benefit sharing, incentives)		1^127^			1
	Biobanking Research (results sharing, benefits sharing, data ownership)			1^128^		1
Real and hypothetical	Clinical Research	Coercion in research participation		1^126^		1
Broad topics:	Clinical Research Ethics and/or Ethics Committees			3^67 133 136^	1^103^	4
Specific topics within:	Clinical Research Ethics	Ethics Committees (composition, functioning, ethical review process)		2^68 108^		5
				2^92 121^		
				1^73^		
		Data sharing			1^84^	1
		Outsourcing, Clinical Research Organisations and Civil Society Organisations		3^90 114 115^		3
		Community stakeholder engagement			1^105^	1
		Informed consent documents and processes		1^109^		1
		Impact of regulatory changesf**		2^76 89^		2
**Subtotal**			**4**	**16**	**6**	**26**
**Primary Research Total**			**17**	**34**	**7**	**58**

*B. Secondary Research*								
Population *Reviews of:*		EC documents		Informed consent documents	Study data / documents	Websites	Journal articles	Total
		Application forms	Governance††					
Completeness, errors, quality		3^85 118 120^			1^110^			4
Payment / compensation for:	Participation	1^99^						2
	Injury			1^64^				
Compliance / adherence with:	Regulations			1^95^				4
	Guidelines		1^123^	1^107^				
	Protocol		1^119^					
Readability				1^86^‡‡				1
Ethics committee:	Registration / accreditation					1^106^		2
	Changes in composition/structure after regulatory changes		1^66^					
Reasons for uninitiated studies			1^96^					1
Registered clinical trials and disease burden						2^139 141^		2
Reporting practices on:	Ethical clearance and/or obtaining informed consent/assent						4^63 71 74 94^	6
	Funding sources and conflicts of interest						1^93^	
	Ethical issues/methods of RCTs; journal editorial policies					1§§^124^		
**Secondary Research Total**								**22**

Some studies were with parents of children.^83 111 116 117^

Studies where India is one of the countries among others, but where some findings specific to India were reported have been included.^74 93 94 121^

*Studies that explored knowledge/comprehension were included here, even when Attitude and Practice components were not studied; some studies not included here that minimally explored or mentioned knowledge/awareness have been included elsewhere.^68 103 127^

†There is only one RCT^116^ in the dataset.

‡This study comprised no lay people, but was categorised as ‘Mixed’ because the population comprised Professionals and Documents.

§Pharmacovigilance studies were excluded in general; this study was included as it was in relation to clinical trials in particular and included views on EC functioning.

¶Studies that explored perceptions (or attitudes) or experiences or practices, or a combination of these, were included here.

**Five other studies also address the impact of regulatory changes.^66 72 78 79 106^

††Governance related documents included meeting minutes, project registers/files, standard operating procedures, site visit monitoring reports, study approval letters.

‡‡One other study^62^ also included readability of informed consent form.

§§Study data included journal articles and website (Clinical Trials Registry-India); could also be categorised within compliance/adherence with guidelines (includes journal editorial policy compliance with international guidelines).

EC, ethics committee; RCT, randomised conrolled trial.

*Primary research (n=58)*: more than half (32/58) were studies exploring knowledge (with or without attitude and practice components) of participants on topics such as information provided to obtain informed consent (primarily with lay participants), clinical trials/research, research ethics and ethics committees (primarily with professional participants), and were mainly quantitative (27/32). Studies that assessed comprehension of the informed consent form or verbal information provision (n=10) were carried out in real (8/10) and hypothetical (2/10) randomised controlled trials (RCTs), clinical trials and cohort studies.

Another large group of primary research studies (26/58) focused on perceptions, experiences and practices/processes on topics such as the extent of patient participation in informed consent discussions, AV recording of consent processes, ethics committees, research governance (eg, data sharing) and the larger clinical trials landscape in India (such as outsourcing, contract research organisations and civil society organisations). Studies employed a wider range of methods (11 quantitative, 13 qualitative studies, 2 mixed methods) and some (9/26) were conducted in the context of a real and/or hypothetical study.

*Secondary research (n=22)*: these studies were all quantitative and were centred around documentary reviews of the quality of application forms submitted to ethics committees, compliance of informed consent documents to guidelines/regulations, and Indian journal articles’ reporting practices on informed consent and ethical approval.

### Narrative synthesis: key findings and research gaps

The findings from included studies were synthesised based on population groups (lay/professional) and key topic areas, with summaries of methodological issues where relevant. Sections A1–A6 and B1 indicated below correspond to those in table 3, which highlights the key findings from the synthesis alongside identified gaps (see online supplemental file 7 for full report of synthesis).

10.1136/bmjgh-2020-004729.supp7 Supplementary data

**Table 3:** Summary of synthesised findings and gaps

Topic	Summary of synthesised findings	Research gaps
**A. Primary research: knowledge (or awareness/comprehension) research (with or without attitudes/perceptions and practice/behaviour (or process) components)**		
A1. Comprehension of the clinical trial/research informed consent form and/or verbal information provision (within specific studies—real or hypothetical): *lay (and some professional) participants* Number of studies tagged to topic=10	Studies were questionnaire surveys that varied in methodological quality, with most deficiencies being in relation to survey instruments and reporting practices. Comprehension regarding a large number of aspects were studied among lay participants and reported to be poor on simple (eg, condition under study)^117^ as well as advanced concepts (eg, randomisation and blinding).^65 116^ Findings were mixed in relation to comprehension of some key concepts such as participant rights—some studies reported participants appeared well aware of their rights,^62 78 82 87^ while others noted superficial rather than detailed understanding (eg, being aware of the voluntary nature of participation but not of freedom to decline participation or withdraw without facing adverse consequences).^116 117^ Comprehension among professional participants (medical and nursing students) was reported as insufficient.^91 104^ Except for one RCT that compared different methods of counselling for informed consent (group and individual; no difference in comprehension found),^116^ there were no other interventional studies aimed at identifying strategies that may help improve informed consent. A critical examination of what may constitute optimal understanding or information provision was lacking. The rationale for assessing comprehension was not always clear—only a few mentioned using the outcome to provide further information to participants on topics in which they had a lower score.	(for A1-A4) Despite a large proportion of studies focusing on knowledge (and attitudes and practices), primarily through questionnaire surveys, it is as yet unclear (a) what aspects of clinical trials/research were often better or poorly understood by lay participants from the informed consent form and verbal information provision, (b) what, if any, aspects of clinical trials/research, research ethics and ethics committees participants (primarily professional) were familiar with. There is a need for cross-cultural adaptations of questionnaires used in other countries and/or the development of locally validated survey tools to assess knowledge and comprehension. Research focused on knowledge should also critically examine and report on (a) the purpose of doing this (eg, whether assessing comprehension of informed consent would change local practice) and (b) what constitutes optimal understanding (among research participants) and optimal information provision. Developing a core information set for minimum baseline information to be conveyed to patients is crucial. There is an immense gap in knowledge regarding interventions that can potentially improve comprehension of research participants in India. Research is also needed on interventions aimed at: improving communication of research terminology in local languages, evaluating current clinical trials/research and research ethics coverage in healthcare students’ curriculum and ways to optimise it, improving knowledge of these topics among healthcare providers and faculty. Qualitative research studies that chart the actual practice of informed consent rather than the reported practice of it are needed. Given the existing large volume of studies on ethics committees, research is needed on interventions that support and optimise the functioning of committees to overcome identified barriers.
A2. Knowledge of and attitudes/perceptions to clinical trials/research more generally (not in the context of specific studies): i. *Lay participants* Number of studies tagged to topic=7 ii. *Professional participants* Number of studies tagged to topic=5	Similar to studies above, the methodological limitations of this group of primarily questionnaire surveys hamper a robust understanding of lay and professional participants’ knowledge and attitudes to clinical trials/research. *Knowledge*: the synthesis of findings suggests limited to poor awareness of clinical trials/research among lay^88 113 134 140^ and professional participants^75 112 131^ (healthcare professionals such as doctors, nurses, counsellors and healthcare faculty and/or students from medicine and pharmacology). There was wide variation in the proportion of lay participants (~25%–60%) who had heard of clinical trials/research^69 113 134^ and lack of familiarity with the English term ‘clinical trial’ among professional participants^112^ and the word ‘research’ or its local translations among lay participants.^127^ Lay and professional groups were unfamiliar with the regulations required for biomedical research and/or clinical trials in particular.^75 100 127 129^ *Attitudes*: studies reported generally positive attitudes towards clinical research and its potential benefits across lay and professional groups.^69 75 113 122 127 131 134^ Lay participants’ concerns revolved around confidentiality, compensation for participation and adverse outcomes, unethical trial conduct and lack of trust in pharmaceutical research.^69 122 127 134^ Professional participants had negative attitudes towards pharma or industry-sponsored studies and expressed support for inclusion of clinical trials in the medical curriculum.^75 131^	
A3. Knowledge, attitudes/perceptions and practices in relation to research ethics (including informed consent): *Professional (and some lay) participants* Number of studies tagged to topic=16	As above, these were primarily questionnaire surveys with methodological limitations that limit the synthesis of participants’ (mostly professional and some lay) knowledge, attitudes and practices in relation to research ethics and informed consent (eg, many studies did not report if participants had prior clinical trials/research training/experience). Studies were primarily with dental and medical students and/or faculty and professionals from clinical research organisations, and some with ethics committee members, investigators and lay participants. *Knowledge*: some studies found poor or limited knowledge (self-reported or assessed) of research ethics and ethical guidelines among professional groups,^77 81 137^ while others reported good knowledge but poor attitudes and practices in relation to some aspects of informed consent and research ethics^81 98 132 137 138^ (eg, some support for fabricating data to improve research outcomes if it did not harm patients and willingness to undertake research rejected by ethics committees). *Attitudes:* there were generally positive attitudes amongst professional participants towards procedural aspects ofinformed consent^81 98 137^ (such as informing patients of risks/benefits and obtaining signatures of participants), but concerns existed amongst lay and professional groups whether the informed consent process and documentation truly protect and inform patients.^67^ ^127^ There was overwhelming support for research ethics education for keystakeholders (health students, researchers, ethics committee members),^81 98^ ^137^ but no research on what, if anything, was currently covered in the medical/dental curriculum. *Practice:* there was wide variation in the reported practice of informed consent and some indication of unsatisfactory practices in relation to research ethics and conduct^77 135^ ^138^ (eg, in relation to carrying out informed consent in local languages, providing a copy of the consent documentation to patients and maintaining accurate patient records for research). There was indication of coercion among professional participants^126^ (medical students) and instances of inadequate informed consent and therapeutic misconception among lay participants.^103^ We do not know what information patients expect to be informed about or what recruiters discuss with patients.	
A4. Knowledge, attitudes/perceptions and practices in relation to ethics committees: *Professional (and some lay) participants* Number of studies tagged to topic=18	Ethics committees were among the most researched topics, primarily through questionnaire surveys, with similar methodological limitations as above (eg, missing information on participant demographics and prior training/experience on relevant topics). Studies were conducted with dental and medical professionals (students and/or faculty), ethics committee members, staff from clinical research organisations and lay participants. *Knowledge*: the synthesis suggests limited knowledge (self-reported and assessed) of ethics committee functioning and composition among medical and dental professionals^81 97 102 135 137^ (eg, on quorum requirements, lay representation and frequency of meetings). Lay participants were unaware of role of ethics committees in protecting patient rights.^127^ *Attitudes:* there was widespread support for the existence and need for ethics committees and ethical review amongst dental professionals,^81 98 137^ but variation in satisfaction (high^67^ to limited^108^ ^135^) regarding ethics committee functioning amongst professional groups (medical and contract research organisation staff). Reported challenges faced by ethics committees (as perceived by contract research organisation staff) included conflicts of interest that compromised their independence and pressures from senior management.^133^ The evolution of stricter regulations and guidelines was described favourably by ethics committee members, but they also felt they were too frequentand too many^76^ ^121^ with numerous challenges in implementing some of the newer regulatory changes^76^ (such as renewal of committee registration). There was overwhelming support for a single national research ethics committee to consider multi-centric trials to prevent ‘ethics committee shopping’ (where investigators went to different committees until they obtained approval) amongst contract research organisation staff^89^ ^108^ but lesser support amongst committee members.^76^ Views on how wide the remit of ethics committees should be varied across professional groups (from monitoring serious adverse events to imparting research ethics education to investigators and conducting ongoing monitoring of trials and on-site visits).^68 101^ ^102 108^ *Practice:* research on ‘practice’ related aspects of ethics committees suggests there were many areas of concern in relation to their functioning and composition^92^ (eg, arbitrariness in member selection and lack of choice in refusing membership amongst those affiliated to institutions), responsibilities^92^ ^101 102^ (eg, some committees undertook monitoring of ongoing trials and on-site visits, while others did not), workload^73^ ^92^ ^102^ ^108^ ^130^ (frequently described as onerous), the ethics review process^73^ ^101^ ^102^ (eg, lack of uniformity in documents and ethical aspects reviewed and guidelines followed) and the dilemmas faced in being expected to align with the international standards for ethical review and the increasing pharmaceuticalisation of society, while also protecting national interests and preventing the perpetuation of existing health and social inequities.^121^	
**A. Primary research: perceptions, experiences, practices/processes**		
A5. Informed consent processes: *lay (and some professional) participants* Number of studies tagged to topic=13 (of which only 5 were focused on topic)	A small group of studies (n=5) explored the processes involved in informed consent, with a further few (n=8) briefly touching on the topic. Only one study^70^ detailed the process of customising the informed consent process to the study population (in an RCT with people with schizophrenia) through feedback from participants/caregivers and then evaluating the process from multiple perspectives. Use of a flip-chart during informed consent and training/ongoing support were found to be useful by participants/study personnel, while research terminology (trial/research, randomisation) was reported as difficult to convey.^70^ *Patient participation in informed consent discussions*: questions asked by parents/guardians of potential child participants (infants) in informed consent discussions varied from 13% to 55% in two studies,^83 111^ with education and higher socioeconomic status reported as associated with asking questions.^83 111 127^ In healthy volunteer studies, concerns raised by participants revolved mainly around the payment than about their own health. *Recruitment process/experience and informed consent process*: one study reported on the involvement of paid middlemen to recruit healthy volunteers for bioavailability/bioequivalent studies, serial participation among volunteers and the informed consent process being a mere formality (as decision to participate was often made prior to that). Contrary to views of family involvement in informed consent, healthy volunteers were mostly unaccompanied and had not informed their families of participation due to concerns about being perceived as selling their bodies for money.^140^ *AV recording of informed consent discussions*: acceptability of and support for AV recordings varied (a third of lay participants refused in a hypothetical study and nearly all agreed in a real vaccine trial^72 83^; a third to two-thirds of investigators were in support.^79 89 133^ Concerns included the increase in time/resources required to carry out AV recordings and the lack of adequate guidance and support.^79 83 89^ Some ethics committees reported reviewing the recordings if there was a need (ie, non-compliance/protocol deviations in the informed consent process).^76^ Some investigators believed that the AV recording of the consent process would improve informed consent^83 89 109^ (eg, by increasing investigator responsibility), with one study reporting that study participants had better comprehension scores after mandatory AV recording of consent process than before.^78^	(for A5) Gaps exist in our understanding of (a) models of informed consent that are tailored to the Indian context (ie, community-family based and/or Western-individual autonomy based; in the context of language diversity, illiteracy, health literacy), (b) informed consent/assent in children’s clinical research (c) informed consent processes across different contexts (industry or investigator led; student-led trials in medical institutions; healthy volunteer studies and vaccine trials), including recruitment interactions with potential participants and (d) The dual role played by many trial recruiters, where they are also the doctor/healthcare provider and the conflicts of interest and therapeutic misconception arising from same. Research examining the usefulness of mandatory AV recordings (eg, how often are they accessed for the purpose that they were made mandatory for) and ways in which existing AV recordings can be used to optimise informed consent are needed.
A6. Bigger picture: *professional (and some lay) participants* Number of studies tagged to topic=20 (of which only 7 were focused on topic)	There were a few (n=7), primarily qualitative, studies that explored the larger landscape within which clinical trials were conducted. Four cross-cutting themes were identified, drawing from other studies (n=13). *Compensation (n=10)*: the synthesis revealed a nuanced discussion among professional and lay participants in relation to compensation for free medicines, for participation and for study-related injuries/serious adverse events. For instance, while lay participants from higher socioeconomic groups felt that the product (vaccine) should be free as it was still being researched, those from lower socioeconomic groups perceived free as inferior or dangerous.^127^ Knowledge of and compliance with national laws and guidelines regarding compensation for clinical trial-related injuries varied among investigators, ethics committee members and sponsors (reported as aware to lacking in clarity) and lay participants (reported as completely unaware).^103 125^ There was lack of uniformity in how and by whom compensation was determined (eg, by ethics committees, sponsor or investigators) and for what purposes (eg, lost wages, travel, participation, injuries or their management),^76 103 125^ with some evidence of healthy volunteers being able to bargain for incentives higher than what was approved by ethics committees.^140^ *Sharing of data, blood/tissue samples, results and benefits (n=3)*: the limited experience of participants (lay and professional) in relation to data sharing amplified their concerns about it.^84^ Despite the small number of studies on the topic, issues were well explored in relation to what is data,^84^ views on sharing of blood/tissue/medical records (lay participants often readily agreed at the start but were more discerning when given further information),^127^ different types of consent for data sharing^84 127^ (eg, blanket/broad, middle or explicit consent), disclosing individual findings following the use of biobanking research^128^ (eg, there was some support for disclosing actionable individual results, while recognising the challenges to the process and contrasts with high-income countries where individual results are usually not shared), sample ownership in biobanking research^128^ (eg, patients’, custodians’ or researchers’) and benefit sharing^127 128^ (eg, giving back to the community, especially when outcomes of studies are commercialised for profits). *Power imbalances (n=17)*: unequal power dynamics were explored across different groups and contexts. These ranged from local issues such as lay members of ethics committees feeling stifled by medics and scientists^92 133^ and paternalistic doctor-patient relationships contributing to therapeutic misconception about clinical trials,^127^ to larger issues such as the lack of correlation between India’s disease burden and its clinical trials,^90 92^ capacity building being more about implementation of agendas set by international pharma companies and procedural efficiency than the nurturing of local innovation and leadership,^114 115^ the exploitation of disadvantaged groups in clinical research^103 105 114 140^ (eg, targeting of recruitment within poor, rural, tribal and unemployed groups), paid healthy volunteers being exploited due to their lower socioeconomic status while also being able to bargain for higher incentives than approved by ethics committees (many viewed trial participation as an alternative career)^140^ and ethical variability and the continuation of a neo-colonialist relationship between the West and India.^109 112 113 121^ The larger issues were highlighted by members of civil society organisations and ethics committee members, but less so by those from the private sector and contract research organisations, who argued against ethical variability across the West and India and felt that clinical trials were relevant to the needs of India.^67 90^ Patient and public involvement was under-researched, except for one study on community engagement.^89^ *CROs, CSOs and the clinical trial industry (n=7)*: some studies provided a detailed account of the growth of CROs in India (with ‘big-pharmaceuticalisation’ used to describe Indian pharma companies’ move from generic drug manufacturing to innovative research), CRO operations and processes employed for recruitment (in the context of healthy volunteers)^140^ and the vital role played by CSOs in changing the regulatory landscape in India^114 115^ (few other studies also explored related topics^140^). CRO staff were critical of reports of malpractice, but saw these as issues within other rather than their own CROs (although there was evidence to the contrary).^115 140^ There was some distrust of pharma-sponsored trials among doctors, ethics committee members and CSO staff,^75 92 114^ while investigators from the private sector (in a study authored by researchers from a pharma company) expressed favourable views regarding pharma-sponsored trials.^67^ CSO members were supportive of RCTs, but lamented the lack of focus on wider ethical issues that went beyond procedural and informed consent focused agendas. Their accounts drew from interpretations of a social justice-based approach to health, while also highlighting an evolution of their views from the purely ideological to the more pragmatic (a move away from dichotomies such as Indian/public-good and foreign/private-bad).^114^	(for A6) Although few in number, existing studies provide rich insights on the Indian clinical trials landscape. Research on real compensation awards, especially for study-related injuries, would help chart out current practice, so that recurrent areas of concern can be addressed. The challenges with the implementation of compensation rules could be explored in future studies, especially in light of the recent NDCT Rules, 2019. Empirical information on participant profiles across a range of clinical trials will help inform debates around the recruitment of vulnerable groups. Similarly, qualitative research on doctor (or recruiter)-patient interactions would provide empirical evidence on aspects of communication that contribute to or strengthen therapeutic misconception in trial recruitment (so that interventions can be developed to optimise communication). The impact of the NDCT 2019 Rules in redressing concerns such as conflicts of interest and power imbalances within ethics committees would need to be examined. Further research, especially qualitative, to expand the scope of discussion on issues of equity and justice in clinical trials in India and the role of social determinants such as gender, poverty, caste and their intersectionality would add to the existing rich but small number of studies on the topic. There is an immense gap in relation to research on patient and public involvement in clinical trials.
**B. Secondary research**		
B1. Documentary reviews Number of studies tagged to topic=23	Documents, primarily sourced from ethics committees (such as informed consent documentation, application forms, meeting minutes, site visits, approval letters) were examined for quality, coverage of issues such as compensation and compliance with legal frameworks and good clinical practice guidelines. Documentary research highlighted inadequate informed consent documentation,^119^ increased workload for ethics committees after the regulatory changes of 2013,^66^ inequities in the distribution of clinical trials, medical colleges and ethics committees across different states in India (reflecting existing health inequalities),^106^ mismatch between India’s disease burden and areas researched in clinical trials,^139 141^ evidence of ‘ethics shopping’ (multicentric studies that had not resolved queries raised by one ethics committee were found to have gained approval at another committee),^96^ inadequate mention of compensation arrangements in ethics committee application forms and informed consent documents^64 99 107 120 125^ (with some indication of improvements over time). Where readability of informed consent forms was examined, it was through Western readability tests.^86 107^ A small group of studies also looked at reporting practices in journals from India, mostly in relation to ethical approval and informed consent, and found that this information was often missing or suboptimal.^63 71 74 94^ Methodological and ethical issues were found to be better reported in the clinical trials registry in India than in journals.^124^	(for B1) Empirical evaluations of the regulatory processes, including number of trial applications submitted for approval per year, numbers approved and disapproved and reasons for the same, will help researchers better understand how regulations are applied to trial applications. Research to develop readability tests in Indian languages may help in improving informed consent forms, which could also be examined for issues beyond compliance with legal frameworks/guidelines (such as whether trial treatments are presented in a balanced manner). Studies on reporting practices of surveys published in Indian journals would help highlight the key methodological issues that can be improved.

AV, audio-visual; CRO, contract research organisations; CSO, civil society organisations; RCT, randomised conrolled trial.

Primary research was synthesised in six key areas (A1–A6). The first four (A1–A4) covered studies that involved comprehension of the informed consent form and knowledge of clinical trials/research, research ethics and ethics committees (where attitudes and/or practices were reported, these were synthesised). Research on informed consent processes (A5) and broader cross-cutting themes that provided a more holistic understanding of the clinical trials industry (A6) were also synthesised. Secondary research (B1) was synthesised based on the type of documents scrutinised (eg, ethics application forms, informed consent documents, journal articles) and the area under investigation (eg, completeness, errors, quality; reporting practices). The number of articles tagged to a given topic includes studies where that topic was the main focus as well as those where the topic was briefly explored. Salient findings from the synthesis are presented below narratively.

#### Primary research

The synthesis (table 3) established that, despite the focus on knowledge-based studies evident in the evidence map (table 2), it was difficult to build a coherent picture of lay and professional participants’ understanding of the topics explored (written/verbal information provision, clinical trials/research, research ethics, ethics committees), primarily due to the methodological (eg, validity of survey instruments) and reporting limitations in studies (A1–A4). Methodological research aimed at developing locally validated tools to assess knowledge will help improve the quality of future studies and facilitate meta-analysis.

Ethics committees (A4) were among the most studied topics (18 studies) and also the source of data in a large volume of studies (16 studies, 8 each with committee members and documents submitted to/produced by committees). Studies highlighted a number of challenges faced by ethics committees^73 92 101 102 108 121 130^ (eg, conflicts of interest, onerous workload, impact of frequent regulatory changes without support for implementation), which would benefit from the development of interventions to support the optimal functioning of ethics committees. Healthcare students were the next most researched group (10 studies).

Research on interventions to optimise comprehension of written/verbal information provision for informed consent (A1) were particularly lacking (except one RCT that compared group and individual counselling and found no difference in comprehension).^116^ While there is some evidence of the difficulties of communicating research terminology (around terms such as research, trial, randomisation) particularly in local languages,^70 112 127^ research is required on interventions to overcome these barriers (A2). There was overwhelming support for education and training on clinical trials/research and research ethics in the curriculum for key stakeholder groups, including healthcare students^75 81 98 131 137^ but we do not know what, if any, aspects of these topics are currently covered in healthcare students’ curriculums so that deficiencies can be identified and addressed (A3).

There is some evidence in relation to the ‘reported’ practice of informed consent^77 126 135 138^ (eg, not conducting informed consent in local languages or indication of coercion among student research participants), but limited^70 83 111 140^ information on the ‘actual’ practice of gaining informed consent, what research participants consider important to know or models of informed consent that are tailored to the local context (A3, A5). Where ‘actual’ practice was examined, it was illuminating—for instance, in healthy volunteer studies, informed consent appeared to be a formality and discussions were centred around payment for participation than risks to volunteers’ health.^140^ Future research on informed consent processes should include an in-depth exploration of the recruitment interaction with potential research participants that delves beyond the questions participants ask, towards the identification and dissemination of good practice, across multiple contexts (eg, consent/assent in trials with children; student-led trials in academic institutions). A good starting point would be to explore if it is feasible, within the current regulatory framework and following strict confidentiality requirements, to use the AV recordings of the consent process more proactively for these purposes, rather than be reviewed only when there are reports of ethical misconduct.^76^ Similarly, the development of core information sets that help define the essential information that participants would like to receive is warranted (A3, A5).

The small group of studies (A6; seven studies) that focused beyond the surface issues around clinical trials provided rich insights into the origins, growth and workings of the clinical trials industry, while placing the industry within the wider regulatory environment and existing health inequities. Four key cross-cutting themes were examined among these primarily qualitative studies (informed by other qualitative/quantitative studies that touched on similar areas):

- Compensation (for study participation, treatment or study-related injuries) was well researched and studies highlighted the need for a nuanced consideration of compensation arrangements^127^ (to account for views such as free treatment being perceived as inferior/dangerous by those from lower socioeconomic groups). It also appeared that compensation determination is fraught with challenges^76 103 125 140^ (such as lack of uniformity in the process and incentives approved by ethics committees being overridden). Studying current practice in relation to actual compensations that have been awarded may help chart out areas of inconsistencies that can be addressed. Also, there appear to be challenges with implementing and complying with the compensation rules, which could be investigated in future studies (no studies were conducted after NDCT Rules 2019).
- Data sharing was explored in a small volume of studies^84 127 128^ that nonetheless provide valuable insights. For instance, lay participants appeared cautious about consent for data sharing after receiving detailed information (despite readily agreeing initially)^127^ and some professional participants supported sharing clinically relevant and actionable results with individuals who contributed to biobanking research, but acknowledged the challenges to this process.^128^
- Power imbalances within the clinical trials/research environment were frequently discussed by professional participants, especially members of ethics committees and civil society organisations. Imbalances of concern included the paternalistic doctor-patient relationship contributing to therapeutic misconception^127^ (where participants perceive unproven trial treatments to be beneficial), the lack of correlation between India’s disease burden and diseases studied,^90 92^ the equation between paid healthy volunteers (exploited due to their lower socioeconomic status) and contract research organisations (with whom the volunteers have bargaining power),^140^ capacity building that does not foster local innovation^114 115^ and the hierarchy between medical and non-medical experts in ethics committees.^92 108 133^ Some of these concerns would benefit from empirical investigation—for instance, studying the doctor-patient interaction in trial recruitment can help delineate the components of communication that contribute to therapeutic misconception. Similarly, research, particularly qualitative, that further explores issues of equity and justice in relation to clinical trial recruitment processes is warranted. Research on patient and public involvement in clinical trials is conspicuous by its absence and should be prioritised to redress some of the power inequities.
- A small group of studies provided nuanced insights into organisations that appear to be at opposite ends of the ethical debates on clinical trials in India—contract research organisations (CROs) and civil society organisations (CSOs).^114 115^ Although critical of ethical malpractice in general, CRO staff were less inclined to acknowledge instances of the same in their own CROs.^115^ CSO representatatives were supportive of clinical trials, felt the need to move away from pitting Indian and/or public sector clinical trials against foreign and/or private sector clinical trials as good versus bad and emphasised the need to focus on wider ethical issues that delve beyond simplistic procedure-based agendas.

#### Secondary research

The synthesis of documentary research (B1) corroborated findings from the synthesis of primary research and reported: inadequacies in informed consent documentation, increased workload for ethics committees particularly after the 2013 regulatory changes, mismatch between clinical trials and India’s disease burden, lack of uniformity in compensation mechanisms and suboptimal clinical trial reporting practices in Indian journals.^64 66 71 74 119 124 125 139 141^ The use of Western readability tests for written information provided in India^62 86^ needs addressing with the development of readability tests in Indian languages. Similarly, while studies on journal reporting practices have focused on the reporting of ethical approval and informed consent, future studies could investigate reporting practices in relation to questionnaire surveys (given their frequent use and methodological/reporting limitations as indicated earlier).

### Consultation exercise

Nine of the 10 individuals approached agreed to participate in the consultation exercise (virtual conferencing group: n=7, one meeting, 1 hour 30 min; telephone: n=1; email: n=1). The consultation group’s recommendations and actions taken were grouped into five key areas as summarised in table 4 (detailed in online supplemental file 5).

**Table 4:** Recommendations from the consultation group and actions taken

Area	Recommendations	Action
1. Improving the manuscript	Change title to better reflect the scope of the review. Ensure better acknowledgement of the rich bioethics literature and lack of grey literature in the review. Incorporate a reflexive section on the authors. Emphasise the value of qualitative research in addressing key research gaps.	Reflexive note in online supplemental file 5; others incorporated in manuscript.
2. Additional analysis and missed literature	Consider impact of the 2013 regulatory changes. Consider impact of studies’ funder/sponsor on the research landscape. Examine four missed articles for inclusion.	Additional analysis undertaken (data extracted for year of data collection and funder). One article met inclusion criteria and was included; others, where relevant, have been mentioned in methods/discussion.
3. Research gaps	There is insufficient empirical information on: Informed consent/assent processes for children in clinical trials/research. Models of informed consent to suit multiple contexts. Issues of equity and social justice in relation to clinical trials. Doctor-recruiter dual role and the arising conflicts of interest. Regulatory processes. Academic trials conducted in medical institutions and vaccine trials. Therapeutic misconception. Questionnaire validation processes.	These gaps have either been highlighted separately within the review or incorporated within existing gaps.
4. Reasons for paucity of research	Lack of funding initiatives to carry out nested studies within clinical trials and related methodological work is a major obstacle for researchers in India. Not all ethical issues are ‘researchable’ and are sometimes better captured through bioethics literature.	Incorporated in discussion.
5. Concerns	Most concerns expressed were in relation to ethics committees: Lack of awareness of principles underpinning clinical research and good clinical practice guidelines among committee members. Non-trial study designs encouraged by committees to avoid institutional liability for serious adverse events in clinical trials. Excessive workloads and undeclared roles and conflicts of interests among members.	Noted here as this is a reflection of the large proportion of studies on ethics committees.

## Discussion

We carried out a scoping review and narrative synthesis of the empirical literature on ethical issues in relation to clinical trials/research in India. We developed an evidence map of 80 studies and synthesised the findings narratively, revealing a wide range of topics investigated and the gaps that exist, with key insights from the consultation group. We found that some topics and populations were more favoured than others—the literature was heavily focused on ‘knowledge’ assessments of participants from lay/professional groups on various topics; ethics committees were examined from multiple angles while also being the source of data in many studies and healthcare students were often research participants. On the other hand, studies that investigated the recruitment-informed consent process, models of informed consent tailored to the Indian context and issues such as equity and justice in the context of clinical trials/research were far fewer in number or absent.

To our knowledge, this is the first systematic scoping review that focuses on empirical research on the ethical aspects of clinical trials/research in one country. Systematic reviews on related aspects (eg, willingness to participate) have tended to combine LMICs together^44^ or included people living in India with those of Indian origin living in other countries.^45^

Our findings indicated that the volume of literature on a given topic was not associated with whether or not it allowed the development of a cohesive synthesis on the topic. We found it challenging to develop a lucid picture of some frequently researched areas such as knowledge on clinical trials/research and research ethics. Given the diversity and scale of the population in India, this could be a reflection of reality, but the numerous methodological limitations and reporting variations, particularly among questionnaire surveys, made it difficult to identify commonalities that may exist. By contrast, although only a small number of studies focused on the wider ethical issues, they provided valuable insights into the workings of the clinical trials/research industry. This may also be because the former group of studies, primarily questionnaire surveys, were likely aiming for breadth but were often compromised methodologically, while the explorations of wider ethical issues were more amenable to qualitative research and successfully provided the depth that was warranted in intense and nuanced debates.

Research gaps were identified on topics that need to be researched (when limited or missing from current literature) as well as topics that need to be ‘better’ researched (when present in literature but requiring methodological/reporting improvements). Given that questionnaire surveys (particularly those exploring knowledge) were the predominant method used, methodological research on developing and validating culturally relevant survey tools and minimum journal reporting standards for surveys would be crucial, drawing from existing guidelines.^144–146^ Small-scale, single-centre surveys may be useful to inform local practice, but consistent use of validated measures and standardised reporting practices are needed to contribute to national policy and practice. Calls to ensure inclusion of research ethics and clinical trials education in the curriculum of healthcare students would be bolstered if research can establish and evaluate the content of aspects that are already covered.

The direct impact of the 2013 regulatory changes on the research landscape are unclear in this review. A few studies investigated professionals’ perceptions of regulatory changes,^76 89^ acceptability and impact of new measures such as the AV recording of consent^72 78 79^ and the impact of changes on ethics committees^66 106^ (latter is examined in-depth in an excluded literature review^147^). It would have been useful to further examine the review findings through the prism of the landmark 2013 regulatory changes, but with a third of the studies not reporting the year of data collection, this was not feasible. It is also important to interpret the findings in light of the continually evolving regulatory landscape in India, with the most recent changes introduced in March 2019 (NDCT Rules).^19^ For instance, some studies raised concerns in relation to the conflicts of interest that compromise the independence of ethics committee members and the hierarchy between medical and non-medical (lay) members of ethics committees, stemming partly from issues such as lack of adequate training for lay members.^92 108 133^ With the NDCT Rules now requiring 50% of members to not be affiliated to the institution in which the committee is based and necessitating mandatory training for ethics committee members,^148^ future studies can investigate if this has redressed some of the concerns around the independence of ethics committees and the power imbalances within. Similarly, Indian regulations on compensation for trial-related injuries are acknowledged as comprehensive and having unique features (eg, the compensation for injuries not related to research),^149^ but it would be crucial to study the challenges in the implementation of these national laws on compensation.

The views expressed by some participants (and authors) of studies in this review that there was an excessive focus on the proceduralism of informed consent is conceivably true in practice and appears well documented,^67 90 101 121^ yet the informed consent process was grossly under-researched. Given the breaches of good practice reported in the past and the routine AV recording of the informed consent interaction, it is notable that only one study^83^ was conducted using this resource. It is unclear if the challenges in undertaking, storing and retrieving AV recordings^150 151^ has a role in their underutilisation for research purposes or if this is due to regulatory restrictions. Opening the black box of the informed consent process in future qualitative research can help optimise comprehension of participants, communication of complex trial-related terminology in local languages and identify aspects of the doctor-patient interaction that contribute towards therapeutic misconception.

Given the lack of established benchmarks for what constitutes optimal information provision for potential clinical trial participants in India or in the West,^152^ researchers could also establish core information sets (information of core importance to convey to patients, drawing from empirical evidence and consensus building approaches.^153^ Patient and public involvement would need to be a central component in such efforts. Interventions to identify informed consent models that are suited to the Indian context (community-family based and/or Western-individual autonomy based) and to specific situations (eg, industry-led and investigator-led trials) are warranted.

It would also be useful to critically consider the topics, populations and methods that we, as researchers, choose to investigate and employ in future studies—for instance, (a) whether the ease of access to healthcare students and ethics committee members and/or its documentation justifies them being frequently researched, especially when they are so unrepresentative of participants in trials or (b) whether assessing comprehension of informed consent information is meaningful without assessing the quality of written and/or verbal information provision that preceeded it. Future research could also address the lack of readability tests in Indian languages, develop interventions to improve ethics committee functioning by overcoming some of the identified barriers and curtail the excessive focus on ‘knowledge’ to redirect efforts on the larger ethical issues to tackle the inequities and imbalances in the clinical trial industry.^90 92 105 112 114 115 121 127 128^ However, if knowledge assessments were to be undertaken, it would be prudent to consider what constitutes optimal understanding among research participants^152^ and whether the outcome of any knowledge assessments can be used to improve the informed consent process or the comprehension of participants locally. The suitability of interventions employed in high-income countries to improve participant understanding in informed consent for research^154 155^ needs to be carefully assessed for India. Qualitative research methods, underused in the range of topics covered in this review, are best suited to investigate the larger issues that require depth of understanding rather than breadth.

The consultation exercise with key stakeholders in India was instrumental in contextualising this scoping review and identifying missed research priorities. A key structural constraint identified in the consultation exercise and evident in the dataset was that most studies were conducted with no to limited external funding. Calling for high-quality studies that span a range of topics to fill the identified gaps would be misguided without appropriate funding mechanisms. Initiatives such as the Medical Research Council’s trials methodology hubs across the UK have been instrumental in improving clinical trial design, conduct and reporting (eg, see final report of trials methodology research carried out over 4 years, 2014–2018, in one of the hubs^156^), with subsequent provisions for initiating trials methodology projects in LMICs.^157^ It is time for international/national funding agencies to consider establishing similar methodology hubs led by researchers in India, with a focus on the ethical conduct of clinical trials. It would be important, however, to ensure that in our pursuit of empirical evidence, we do not downplay the vital role played by other forms of evidence and catalysts for change, given that not all ethical issues are amenable to being researched.

### Limitations

Despite our best efforts, we may have missed some relevant journal articles and studies included in books. However, if missed articles reflected the patterns of published research included in this review, it is likely that they would not substantially alter our synthesis and conclusions. A decision to only include peer-reviewed research also meant we did not seek out grey/unpublished literature^158 159^ (although condensed publications from them, if any, are included^103^). Some of the topics we excluded may have helped contexualise our findings. For instance, we included studies on research ethics but excluded those on medical/clinical ethics—an associated topic of interest that requires a separate review.

While the review has helped underline the gaps in the existing literature, it is not exhaustive and cannot claim to have identified all gaps. It also cannot prioritise the identified gaps in a meaningful way and is limited in identifying key topics that are completely absent or of importance to key stakeholders. Designing and conducting the review with the input of researchers in India from conception stages may have resulted in a different focus and outcome. Our intention was that the critical input of key stakeholders at the consultation phase helped focus the review and overcome some of the shortcomings. A locally led priority-setting exercise, informed by this review, to determine pressing concerns that warrant empirical investigation would be an ideal next step.

## Conclusion

This systematic scoping review is the first attempt at summarising peer-reviewed empirical research on topics related to the ethics of clinical trials/research in India. The review demonstrates that while a wide range of topics have been studied in India, the focus is largely on assessing knowledge levels across different population groups. This is a useful starting point, but fundamental questions remain unanswered about the recruitment and informed consent process, such as the doctor-patient interaction, and the larger issues of equity and justice that dominate the clinical trials/research landscape.

The evidence map and narrative synthesis are meant to be a starting point for discussions on future research directions, to be used in ways that benefit the research community and patient population and contribute towards the ongoing efforts within India to improve the clinical trials/research ecosystem. A priority-setting exercise that could be informed by this review, led by researchers in India, would be an ideal next step, alongside funding mechanisms that support researchers based in India to undertake research in priority areas in clinical trials/research methodology and ethics.

## Data Availability

All analysed data relevant to this study are included in the manuscript or uploaded as supplementary information. The dataset on which this work is based consists of articles already available within the published literature.
