# Supplementary material for: What empirical research has been undertaken on the ethics of clinical research in India? A systematic scoping review and narrative synthesis
Source: BMJ Glob Health. 2021 May 18;6(5):e004729. doi: 10.1136/bmjgh-2020-004729 (PMC8137180; doi:10.1136/bmjgh-2020-004729)
Supplement: Supplementary data [file bmjgh-2020-004729supp001.pdf]

BMJ Publishing Group Limited (BMJ) disclaims all liability and responsibility arising from any reliance placed on this supplemental material which has been supplied by the author(s)


**Supplementary file 1: Figure - Clinical trials approved in India by year and the evolution of research on the ethics of clinical trials in India mapped against key regulatory developments**

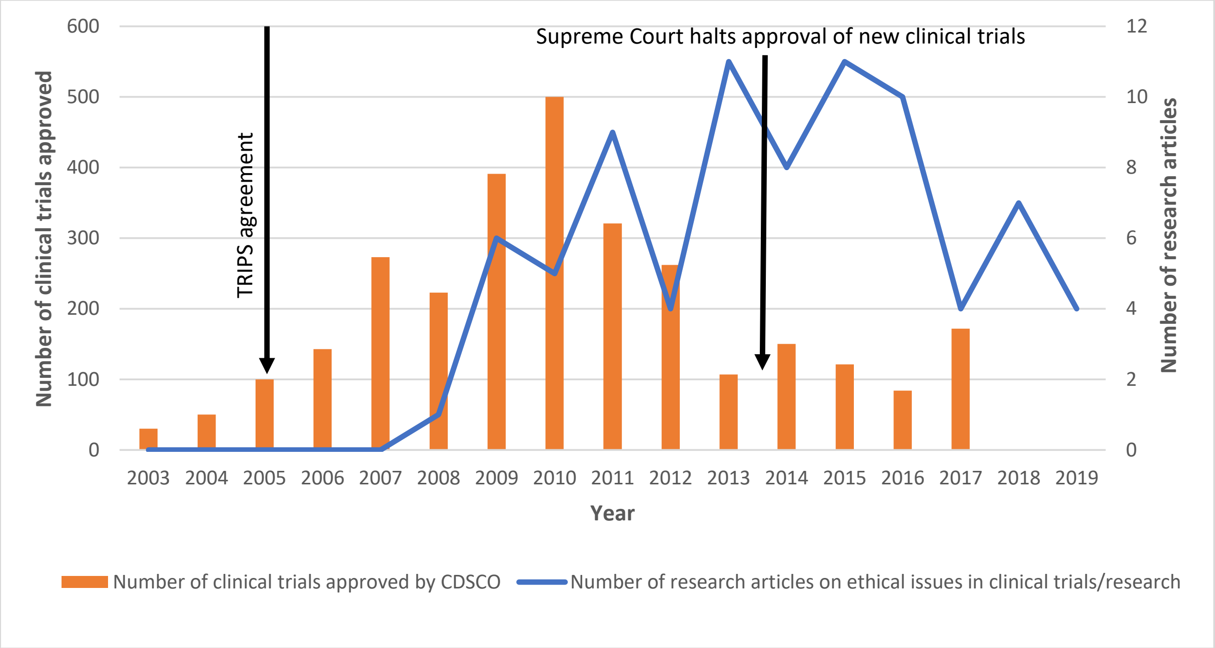


Source: Data on number of trials approved by CDSCO (Central Drugs Standard Control Organisation) is taken from multiple articles; 2003-2008;[2] 2009-2014;[3] 2015-2017[31]
