# Supplementary material for: What empirical research has been undertaken on the ethics of clinical research in India? A systematic scoping review and narrative synthesis
Source: BMJ Glob Health. 2021 May 18;6(5):e004729. doi: 10.1136/bmjgh-2020-004729 (PMC8137180; doi:10.1136/bmjgh-2020-004729)
Supplement: Supplementary data [file bmjgh-2020-004729supp003.pdf]

Supplementary file 3: Inclusion criteria for identification of eligible studies

**Setting**	India (other South Asian countries included in search, but not in any further steps reported)
**Population**	Any stakeholder groups<br>• Lay – patients/patients' guardians, public, CT/cohort study participants;<br>• Professional – healthcare/research faculty, students or practitioners, members/staff of ethics committees or regulatory/governmental agencies<br>• Other – relevant documents
**Phenomenon of interest**	Any ethical aspects of conducting clinical trials/research in India (e.g. informed consent, scientific misconduct, research governance, ethics committees and approvals, good clinical practice)
**Study design**	Primary/secondary research of any design conducted on human participants (including observational, experimental, quasi-experimental, randomised controlled trials, qualitative, mixed methods)
