# Supplementary material for: What empirical research has been undertaken on the ethics of clinical research in India? A systematic scoping review and narrative synthesis
Source: BMJ Glob Health. 2021 May 18;6(5):e004729. doi: 10.1136/bmjgh-2020-004729 (PMC8137180; doi:10.1136/bmjgh-2020-004729)
Supplement: Supplementary data [file bmjgh-2020-004729supp004.pdf]

Supplementary file 4: Medline search strategy

Database: Ovid MEDLINE(R) Epub Ahead of Print, In-Process & Other Non-Indexed Citations, Ovid MEDLINE(R) Daily and Ovid MEDLINE(R) <1946 to Present>

Search Strategy:
----
1 exp Informed Consent/ (39822)
2 consent*.tw. (66248)
3 (informed adj2 (decision* or choice*)).tw. (9675)
4 exp Comprehension/ (11748)
5 exp Awareness/ (18083)
6 exp bioethical issues/ or exp bioethics/ or exp complicity/ or exp "conflict of interest"/ or exp ethics committees/ or exp ethics, institutional/ or exp ethics, professional/ or exp ethics, research/ or exp professional misconduct/ (106864)
7 scientific misconduct.tw. (863)
8 therapeutic misconception.tw. (216)
9 exp Disclosure/es, lj [Ethics, Legislation & Jurisprudence] (4428)
10 disclos*.tw. (70367)
11 research governance.tw. (246)
12 good clinical practice.tw. (1424)
13 exp Confidentiality/ (50771)
14 *Health Knowledge, Attitudes, Practice/ or *patient education as topic/ (86839)
15 ((understand* or knowledge or perception* or comprehend* or comprehension or awareness) adj12 (barrier* or research or study or studies or trial or trials)).tw. (292135)
16 (information adj3 (patient* or volunteer* or participant* or recruit or recruits) adj3 (study or studies or research or trial or trials)).tw. (1179)
17 or/1-16 (661221)
18 exp Clinical Trial/ (831342)
19 exp Clinical Trials as Topic/ (322063)
20 exp drug approval/ or exp drug evaluation/ or exp feasibility studies/ or exp pilot projects/ (217419)
21 exp Human Experimentation/ (12631)
22 exp Research Subjects/ (15759)
23 ((participa* or tak* part or enrol* or volunteer* or recruit* or subject*) adj7 (trial or trials or research or study or studies)).tw. (552279)
24 ((patient* or candidate*) adj7 (trial or trials or research or study or studies) adj7 (choose* or chosen or choice* or select*)).tw. (20782)
25 (exp Patient Participation/ or *patient selection/ or *volunteers/ or *health personnel/ or *research personnel/) and (trial or trials or study or studies or research).tw. (32320)
26 researcher subject relations/ (1086)
27 *drug industry/ (19424)
28 or/18-27 (1709573)
29 exp bangladesh/ or exp bhutan/ or exp india/ or exp nepal/ or exp pakistan/ or exp sri lanka/ (126661)
30 (bangladesh or bhutan or india or nepal or pakistan or sri lanka).tw. (112390)
31 exp Developing Countries/ (71257)
32 exp Contract Services/ (12492)
33 outsour*.tw. (1525)
34 contract research organi#ation.tw. (76)
35 or/29-34 (243873)
36 17 and 28 and 35 (3178)
37 letter/ (992989)
38 editorial/ (452191)
39 news/ (186037)
40 exp historical article/ (387093)
41 Anecdotes as topic/ (4934)
42 comment/ (705965)
43 (letter or comment*).ti. (127313)

44 or/37-43 (2228855)
45 36 not 44 (2827)
46 exp animals/ not humans/ (4581034)
47 exp Animals, Laboratory/ (836666)
48 exp Animal Experimentation/ (8778)
49 exp Models, Animal/ (516385)
50 exp rodentia/ (3100283)
51 (rat or rats or mouse or mice or rodent*).ti. (1315778)
52 or/46-51 (5412057)
53 45 not 52 (2811)

Paramasivan S, *et al. BMJ Global Health* 2021; 6:e004729. doi: 10.1136/bmjgh-2020-004729
