# Supplementary material for: What empirical research has been undertaken on the ethics of clinical research in India? A systematic scoping review and narrative synthesis
Source: BMJ Glob Health. 2021 May 18;6(5):e004729. doi: 10.1136/bmjgh-2020-004729 (PMC8137180; doi:10.1136/bmjgh-2020-004729)
Supplement: Supplementary data [file bmjgh-2020-004729supp005.pdf]

Supplementary file 5

A. Members of the consultation group

**Names (in alphabetical order)**	**Role and Organisation**	**Method of consultation**
1. Dr Amar Jesani	Co-founder of the Forum for Medical Ethics Society; Editor Indian Journal of Medical Ethics; Faculty member, Centre for Ethics, Yenepoya University, Mangalore, India	Virtual group meeting*
2. Dr Anant Bhan	Researcher in Global health and bioethics; Adjunct Professor, Centre for Ethics, Yenepoya University, Mangalore, India; Former President, International Association of Bioethics; Lead, Sangath-Bhopal, India	Virtual group meeting
3. Professor Gagandeep Kang	Professor of Microbiology, Wellcome Trust Research Laboratory, Division of Gastrointestinal Sciences, Christian Medical College, Vellore, India	Virtual group meeting
4. Dr Manjulika Vaz	Lecturer, Health and Humanities, St. John's Medical College, Bangalore, India	Virtual group meeting
5. Professor Nithya Gogtay	Professor and Head, Department of Clinical Pharmacology, Seth GS Medical College and King Edward Memorial Hospital, Mumbai, India	Telephone
6. Dr Rashmi Rodrigues	Associate Professor, Department of Community Health, St. John's Medical College, Bangalore, India	Virtual group meeting
7. Ms Sarojini Nadimpally	Executive Director, SAMA Resource group for women and health, New Delhi, India	Virtual group meeting
8. Professor Urmila Thatte	Emeritus Professor, Department of Clinical Pharmacology, Seth GS Medical College and King Edward Memorial Hospital, Mumbai, India	Virtual group meeting
9. Dr Vijay Gopichandran	Assistant Professor, Community Medicine, ESIC Medical College, Chennai, India	Email

***** The virtual group meeting was held on October 23rd, 2020

B. Reflexive note on the systematic scoping review's authors: The authors of this paper are qualitative researchers (SP, JW, LR, NM, ARe, JD), systematic reviewers (PD, AR), bioethicists (JI, RH, SS) and clinician-researchers (JR, JB). SP was born, raised and educated first in India and then in the UK, with brief clinical work experience in India and research experience primarily in the UK. JR and SS have carried out research in India. All other authors primarily conduct research in the UK.

Amongst the authors, SP, JI, JR and JD and amongst the consultation group, AJ, AB, NG, SN and UT are involved in a recently-funded feasibility study (MRC-NIHR Trials Methodology Research Partnership global health pump-priming grant) on optimising informed consent in clinical trials in India, as co-applicants, collaborators or advisory panel members.

C. Summary of key recommendations from the consultation exercise

	**Recommendations, explanations and current concerns raised by the consultation group and the actions taken thereof in the manuscript**
1. Improving the manuscript	• Title: Previous title ('Ethical issues in clinical trials in India: a systematic scoping review and narrative synthesis to map the quantitative and qualitative evidence and identify research priorities') was considered problematic as it suggested that the review was identifying ethical issues in clinical trials in India, which was not the aim of the authors. Also, the review was broad and included 'clinical research and clinical trials' but the title only mentioned 'clinical trials'. ***Action:*** *Title was changed to 'What empirical research has been undertaken on the ethics of clinical research in India? A systematic scoping review and narrative synthesis to map the evidence.'*

	• Bioethics literature: Ensure better acknowledgement of the bioethics literature that includes reflective, narrative and philosophical debates, as well as case studies of ethical misconduct, which have not been covered in this scoping review, but have been instrumental in changing the regulatory landscape in India. ***Action:*** *Acknowledged in the introduction.*<br>• Grey literature: Acknowledge limitations of not including grey literature, including studies that may have been reported in books. ***Action:*** *Mentioned in the limitations.*<br>• Reflexivity: Include a note on reflexivity to ensure lead author's views regarding own background as expressed at the meeting are presented to the readers. ***Action:*** *Included above in this supplement.*<br>• Qualitative research: The role of qualitative research in providing rich empirical evidence to address some of the gaps needs to be strengthened in the manuscript. ***Action:*** *Further emphasised in the discussion.*
2. Additional analysis to undertake for this scoping review and published or unpublished literature to consider	Analysis:<br>• Impact of 2013 regulatory changes: Consider the impact of the regulatory changes in relation to ethics committees (i.e. to examine if the regulatory changes made a difference to how committees operated before and after 2013) and if there are any significant changes in the nature/type of studies in the scoping review dataset or in the findings more generally before and after 2013. ***Action:*** *To facilitate this, additional analysis undertaken involved extracting the year of data collection from studies, but this demonstrated that a large proportion of studies did not report the year of data collection; this has now been included in the results and limitations sections. A review paper (excluded from this scoping review) that describes the impact of the 2013 regulatory changes on ethics committees in detail[147] has been included in the discussion section of this scoping review.*<br>• Study funder/sponsor: Consider whether it is possible to examine the studies based on who funded/sponsored the study, as the type of studies conducted or the issues explored may vary based on whether sponsored by academic centres or not. ***Action:*** *Additional analyses involved extracting and analysing each paper's corresponding author's institution (academic or not), declarations of source of funding and conflicts of interest – included in the results and discussion sections.*<br>Literature<br>• Published research:<br>a. A qualitative study on ethical issues in the recruitment of healthy volunteers was highlighted as missing from the scoping review. ***Action:*** *This has now been included in the manuscript results.[140]*<br>*b.* Some studies that report on the Clinical Trials Registry India data were highlighted during the consultation exercise. These provide valuable information but audits of the Clinical Trials Registry of India were excluded where they reported the number of trials registered per year[40] or highlighted the deficiencies in the data[41] (and included if they were linked to an ethical issue[139,141]). ***Action:*** *This has now been further clarified in the methods.*<br>• Grey literature: Two studies undertaken by SAMA, New Delhi were mentioned during the consultation exercise and later sent to the scoping review lead (an unpublished comparative study examining compensation mechanisms in seven countries including India[158] and a full unpublished report[159] of a published and included study.[103] ***Action:*** *These have been mentioned in the limitations section.*
3. Research gaps	• Children in RCTs: There is a notion that parents would be less inclined to allow their children to participate, yet experience on the ground suggests that parents are willing to allow their children to participate. Research questions to consider: What drives parents to allow children to participate in trials? What are the issues in the consenting process? How is assent taken care of? ***Action:*** *Informed consent/assent in relation to children's participation in clinical research has been included as a research gap.*<br>• Informed consent:

Paramasivan S, *et al. BMJ Global Health* 2021; 6:e004729. doi: 10.1136/bmjgh-2020-004729

	○ It is unclear how written consent is operationalised in a country like India where a large proportion of the population is illiterate or not literate in the language of the consent form. Research questions to consider: How is written consent obtained in the context of multiple languages, illiteracy and healthy literacy in India? Does picture-based informed consent work better than video consent? ***Action:*** *Expanded section on models of informed consent in gaps identified.*<br>○ There is a need to develop models of informed consent that are based on communitarian models suited to the Indian context rather than the Western libertarian/autonomy models that currently inform our regulations/guidelines. ***Action:*** *This has been further emphasised in the review.*<br>• Recruitment process: There is a need to develop a sound empirical holistic understanding of the entire continuum of the recruitment process, that takes into account issues of equity and fairness as well as social determinants such as gender, poverty, caste and class and their intersectionality. Most of the clinical trial recruitment happens from the hospitals where the health care providers are themselves the researchers; there exists a strong conflict of interest, which needs to be explored. ***Action:*** *Further emphasised in the review.*<br>• Regulatory processes: There is a lack of empirical evaluations of the regulatory processes (e.g. number of trial applications submitted for approval per year, the numbers approved and disapproved, and reasons for the same). ***Action:*** *Included as a gap in the review.*<br>• Ethics of academic clinical trials within medical institutions: Many academic clinical trials happen in medical institutions, including those conducted by post-graduate residents, but they are rarely researched and scrutinized. ***Action:*** *Student-led clinical trials included in gaps.*<br>• Vaccine trial acceptability: There is little empirical evidence as to how vaccine trials are perceived by people and the ethical consideration that inform vaccine developers. ***Action:*** *Included within gaps in the review.*<br>• Therapeutic misconception: Most trial participants experience therapeutic misconception at some level. There is a need to better understand this phenomenon. ***Action:*** *Further emphasised in review.*<br>• Validated questionnaires: There is a need for cross-cultural adaptation (as opposed to translation) of validated questionnaires/tools from other countries, which is sometimes not allowed. For instance, in a study to evaluate osteoarthritis with patient-reported outcome measures, a validated questionnaire asked if the patient can put on and take off stockings, which is not relevant in the Indian context; when asked if that can be changed to sitting or getting up from an Indian toilet, the request was refused. It is likely that similar issues exist for questionnaires used in studies in this review. ***Action:*** *Included as a gap in the review.*
4. Explanations for some of the findings	• Reasons for paucity of RCTs (and nested RCTs or other types of nested studies) in the dataset: Systematic empirical research requires financial support for academics, which is not easily available for researchers in India. Most researchers are expected to carry out research alongside their usual clinical or other duties, and under those circumstances, it is difficult to do research that goes beyond explorations of knowledge and perceptions. Funding and resource constraints mean that although a number of researchers, including those working in the rural areas, are interested in conducting empirical work in relation to informed consent and other ethical issues, their interests often stop at ideation. Many research groups, especially in academic medical centres and government medical colleges, have had to avoid clinical trials as they would be liable to pay compensation for serious adverse events, which they do not have allocated funds for. Drawing from personal experiences, ethics committees have been known to ask investigators to redesign their study, such that it is not a clinical trial, as not many institutions have the funds to provide compensation if necessary. ***Action:*** *Need for funding emphasised in the discussion.*<br>• Some ethical issues are simply not 'researchable' – for instance, corruption and exploitation are difficult to research, but are well captured in the bioethics literature. ***Action:*** *Acknowledgement of the same in the discussion/conclusion.*
5. Concerns	• Ethics committees: Key concerns expressed were in relation to ethics committees, in line with the large number of studies on the same. Concerns revolved around the following issues:

	○ Lack of awareness among ethics committee members regarding good clinical practice guidelines and basic principles underpinning clinical research despite training provision for committee members over many years, making it challenging to assess the nuances related to clinical trials regarding risk minimisation or participant protection. With this being the case in trained ethics committees, there was concern about what may transpire in the case of ethics committees in more remote locations functioning without training.<br>○ Ethics committees sometimes request investigators to opt for non-trial designs, to avoid institutional liability for compensation if necessary (as outlined in section above).<br>○ Absence of declaration of roles and conflicts of interest by ethics committee members.<br>○ Increased workload for ethics committee members, which impedes their ability to examine all the relevant aspects in detail.
