# Supplementary material for: What empirical research has been undertaken on the ethics of clinical research in India? A systematic scoping review and narrative synthesis
Source: BMJ Glob Health. 2021 May 18;6(5):e004729. doi: 10.1136/bmjgh-2020-004729 (PMC8137180; doi:10.1136/bmjgh-2020-004729)
Supplement: Supplementary data [file bmjgh-2020-004729supp006.pdf]

Supplementary file 6: Research aims, settings and methods of individual studies

Study	Research aims and setting	Research methods
**A. Primary research: Knowledge (or awareness/comprehension), attitudes (or perceptions), practice (or behaviour)**		
• **Studies on the comprehension of the informed consent form and/or verbal information provision**		
Arora et al, 2011[62]	To assess comprehension of ICF/IC among participants in a first-in-human study of a novel drug in healthy male volunteers; Chandigarh, India	Questionnaire survey; n=50
Bhansali et al, 2009[65]	To assess comprehension of ICF/IC among patients invited to participate in a phase 3 multi-centric trial of a novel lipid lowering agent; Chandigarh, India	Questionnaire survey; n=42
Figer et al, 2017[78]	To assess comprehension of ICF/IC among participants in a Phase 2/3 rabies monoclonal antibody trial, before and after introduction of mandatory audio-visual recording of IC process in 2013; Mumbai, Maharashtra, India	Questionnaire survey; n=38
George et al, 2018[80]	To assess comprehension of ICF/IC in hypothetical RCTs among adult in-patients with non-organic psychiatric disorders and among their key relatives; Vellore, Tamil Nadu, India	Questionnaire survey; n=32 (14 patients; 18 relatives)
Gota et al, 2018[82]	To assess comprehension of ICF/IC among patients enrolled in Phase 1, 2 or 3 interventional studies; Mumbai, Maharashtra, India	Questionnaire survey; n=200
Joglekar et al, 2013[87]	To assess comprehension of ICF/IC among participants in a cohort study aimed at estimating HIV incidence in a high-risk population; Pune, Maharashtra, India	Questionnaire survey; n=1334
Kamath et al, 2014[91]	To assess comprehension of ICF/IC among medical students invited to participate in a hypothetical anti-malarial drug; South India	Questionnaire survey; n=155
Nambiar et al, 2012[104]	To assess comprehension of ICF/IC among nursing trainees participating in a tuberculosis exposure and latency cohort study; Vellore, Tamil Nadu, India	Questionnaire survey; n=138
Sarkar et al, 2009[117]	To assess comprehension and recall of ICF/IC among parents/guardians of a birth cohort of children from urban slums participating in a diarrhoeal surveillance study; Vellore, Tamil Nadu, India	Questionnaire survey; n=368
Sarkar et al, 2010[116]	To assess comprehension of ICF/IC among parents of children from rural pre-schools participating in an RCT of nutritional supplementation, randomised to receive group or individual counselling for IC; Kaniyambadi, Vellore, Tamil Nadu, India	RCT employing questionnaire survey; n=118 (from 16 rural pre-schools)
• **Studies on Knowledge, Attitudes, Practices in relation to clinical trials/research, research ethics, ethics committees**		
Bhowmick et al, 2014[130]	To assess knowledge, attitudes and practice of ethics committee functioning among ethics committee members; Kolkatta, West Bengal, India	Questionnaire survey; n=30 (from 10 ethics committees)
Burt et al, 2013[69]	To study knowledge and perceptions of clinical research among general public; New Delhi, India	Questionnaire survey; n=175 (from eight public locations)
Choudhury et al, 2016[75]	To assess knowledge and perceptions of clinical trials among doctors from government medical colleges; West Bengal, India	Questionnaire survey; n=133 (from three medical colleges)
Deolia et al, 2014[77]	To assess knowledge, attitudes and behaviour pertaining to research ethics among dental professionals in a private dental institute; South India	Questionnaire survey; n=213
Dhodi et al, 2013[131]	To assess knowledge, attitudes and practices towards clinical research among medical students and teachers; Mumbai, Maharashtra, India	Questionnaire survey; n=395
Gopinath et al, 2014[81]	To assess knowledge and attitudes about research ethics and ethics committees among dental faculty; Chennai, Tamil Nadu, India	Questionnaire survey; n=81

Hussain et al, 2019[132]	To assess knowledge, attitudes and practice regarding informed consent process in biomedical research in postgraduate medical students in a private medical college; Karnataka, India	Questionnaire survey, n=114; Group discussions, n=2 (with 10-12 participants each)
Londhey et al, 2015[97]	To assess awareness of ethics committee composition and functioning among medical teachers; Mumbai, Maharashtra, India	Questionnaire survey; n=180
Joshi et al, 2012[88]	To explore awareness, perceptions of and attitudes towards participating in clinical trials among general public; Pune, Maharashtra, India	Focus group discussions and interviews; n=24 (7 trial participants; 17 non-trial participants)
Joshi et al, 2013[134]	To assess awareness, perceptions and attitudes toward clinical trials and their views on methods to create awareness among general public; Pune, Maharashtra, India	Questionnaire survey; n=240 (40 trial participants; 200 non-trial participants)
Krishna et al, 2014[140]	To examine the relationship between contract research organisations (CRO) and healthy volunteers and the recruitment process in relation to bioavailability and bioequivalent studies; Hyderabad, Telangana, India	Case study of one contract research organisation comprising: Interviews (8 CRO staff); group discussions (n=50 healthy volunteers); observations of informed consent discussions (n=40)
Mallela et al, 2015[98]	To assess knowledge and attitudes about research ethics and ethics committees among dental faculty; North India	Questionnaire survey; n=942
Meenakumari et al, 2010[100]	To evaluate awareness of clinical trials among pharmacy undergraduate and postgraduate students; Manipal, Karnataka, India	Questionnaire survey; n=102
Mishra et al, 2018[101]	To examine awareness of ICMR's ethical guidelines, privacy-relation obligations and experiences in implementing ethics guidelines among ethics committee members; New Delhi, India	Interviews; n=19
Mohammad et al, 2011[135]	To assess knowledge, attitudes and practices of healthcare ethics among medical professionals in a government teaching hospital; Aligarh, Uttar Pradesh, India	Questionnaire survey; n=172
Nadig et al, 2011[102]	To assess knowledge, attitudes and practices pertaining to ethics review and ethical guidelines among ethics committee members; South India	Questionnaire survey; n=29 (from 11 ethics committee)
Ramanaik et al, 2015[112]	To explore knowledge and perceptions of clinical trials (with a focus on HIV vaccine trials) among frontline health service providers working with female sex workers and men who have sex with men; Bellary, Belgaum, Bangalore in Karnataka, India	Interviews; n=50
Reddy et al, 2013[137]	To assess knowledge and attitudes about research ethics and ethics committees among dental faculty; Bhimavaram, Andhra Pradesh, India	Questionnaire survey; n=100
Rodrigues et al, 2013[113]	To assess knowledge regarding knowledge regarding research among HIV-infected individuals; Bangalore, Karnataka, India	Questionnaire survey; n=173
Sridharan et al, 2016[122]	To assess knowledge of clinical trials from semi-urban and rural populations in India; India	Questionnaire survey; n=400
Vittalrao et al, 2018[129]	To assess awareness of clinical trials among medical undergraduate students; Manipal, Karnataka, India	Questionnaire survey; n=257
Vyas et al, 2019[138]	To assess knowledge, attitudes and practices regarding informed consent for research purposes among postgraduate resident doctors; Mumbai, Maharashtra, India	Questionnaire survey; n=100
Thatte et al, 2009[125]	To assess knowledge of compensation clinical trial related injury and among various stakeholders and to review policies on the same; India	Questionnaire survey, n=80 (30 investigators, 23 ethics committee members, 27 sponsors); Interviews, n=14 (3 investigators, 6 ethics committee members, 5 sponsors); Documents, n=119 (informed consent documents)

A. Primary research: Perceptions, experiences, practices/processes in relation to clinical trials/research, research ethics, ethics committees

Bindra et al, 2010[67]	To explore perceptions on research ethics among investigators; India	Questionnaire survey; n=29
Brahme et al, 2009[68]	To study the profile and role of ethics committee members in health and research organisations; Pune, Maharashtra, India	Questionnaire survey; n=52 ethics committee members from 12 committees
Chatterjee et al, 2015[70]	To assess feasibility of informed consent procedure in an RCT for people with schizophrenia, from the trial, research and participant perspectives; Tamil Nadu, Maharashtra and Goa, India	Focus group discussions, n=6; consent interviews and participant feedback on IC process, n=332
Chauhan et al, 2015[72]	To explore acceptability of audio-visual recording of the IC process in a hypothetical study and reasons for refusal; Keezhputhupattu, Tamil Nadu, India	Structured interviews; n=150
Chenneville et al, 2016[73]	To assess perceived capacity of medical school ethics committees through ethics committee members and delineate areas for improvement; West India	Research Ethics Committee Quality Assurance Self-Assessment Tool filled by member secretaries of two ethics committees, n=2; Interviews with committee members, n=6
Davis et al, 2017[76]	To explore perceptions of the 2013 regulatory changes for clinical research among ethics committee members; South and West India	Questionnaire survey; n=25 members from 25 ethics committees
Ganguly et al, 2016[79]	To describe the newly introduced audio-visual recording of the IC process for clinical trials and the perceptions of investigators and trial participants of the same; Gujarat, India	Observations of audio-visual recordings of IC process, n=5; Interviews, n=8 (3 investigators and 5 trial participants)
Gupta et al, 2018[83]	To examine the audio-visual consent process during a Phase 3 rotavirus vaccine trial in healthy infants and parent/guardian participation in the consent process; Chandigarh, India	Audio-visual recordings of consent process; n=100
Hate et al, 2015[84]	To examine key stakeholders' perspectives on data sharing in the context of research involving women and children; Mumbai, Maharashtra, India	Interviews, n=22; Focus groups, n=44 in four focus groups (researchers, managers, research participants, ethics committee members)
Jadhav et al, 2013[133]	To understand perceptions regarding the ethics of clinical research among clinical research professionals; India	Questionnaire survey; n=34 (27 sponsor/contract research organisation staff; 6 ethics committee members; 1 investigator)
Kadam et al, 2016[89]	To assess perceptions of the 2013 regulatory changes for clinical research among clinical trial investigators; India	Questionnaire survey; n=73
Kamat, 2014[90]	To elicit perspectives of stakeholders regarding media representation of their work and ethical issues arising from their engagement in clinical trials; Bangalore, Karnataka and Hyderabad, Telangana in India	Interviews; n=42 (3 sponsors, 7 contract research organisation executives, 19 investigators, 13 ethics committee members)
Kandhari et al, 2013[92]	To provide insights into the structure and functioning of ethics committees from the perspective of ethics committee members; New Delhi, India	Interviews; n=17
Nadimpally et al, 2017[103]	To explore perceptions of clinical trials among trial participants and key informants; Gujarat, Maharashtra, New Delhi, Andhra Pradesh in India	Interviews; n=36
Newman et al, 2015[105]	To elicit perspectives and experiences of key informants involved in community stakeholder engagement activities, in the context of previous HIV vaccine trials in four countries, including India; Chennai, Tamil Nadu, India	Interviews, n=93 interviews; Focus groups, n=140 in 21 focus groups
Parikh et al, 2011[136]	To assess the perceptions regarding the clinical drug trial industry among various stakeholders; India	Questionnaire survey; n=181 (clinical research coordinators/assistants, investigators, managers, directors)

Patel et al, 2016[108]	To explore perceptions regarding the ethical review process and performance of ethics committees among clinical research professionals; India	Questionnaire survey; n=385
Patel et al, 2016[109]	To understand perceptions regarding ethical standards and issues in clinical trials in Indian among clinical research professionals; India	Questionnaire survey; n=385
Rajaraman et al, 2011[111]	To assess extent of participation during informed consent process among parents providing consent for children's participation in an observational tuberculosis study; Palamaner, Chittoor, Andhra Pradesh, India	Observation notes on questions asked by parents during informed consent process; n=4382
Sariola et al, 2015[115]	To explore perceptions of contract research organisations' staff regarding changes in the clinical trial industry since 1995 and 2005, outsourcing of clinical trials to India and models of collaborations; Bangalore, Karnataka; Mumbai, Maharashtra; New Delhi, India	Interviews; n=25 (clinical research assistants, managers, protocol writers, quality assurers, statisticians, CEOs)
Sariola et al, 2019[114]	To explore the role of civil society organisations, academic and public health researchers and health activists in changing the regulations for clinical trials in India; India	Interviews; n=25 (academic public health and medical researchers, health activists)
Simpson et al, 2015[121]	To identify the tensions that emerge for ethics committee members as the capacity to conduct credible ethical review of clinical trials is developed across three countries including India	Interviews; n=14 ethics committee members from India
Vaz et al, 2015[127]	To explore the perceptions, motivations and concerns of the public with respect to participation in clinical trials and biobanking-related research; Bangalore, Karnataka, India	Interviews; n=14
Vaz et al, 2018[128]	To understand views on the ethics of biobanking research among ethics committee members and medical researchers; Bangalore, Karnataka, India	Interviews; n=43 (21 ethics committee members and 22 researchers)
Vaidya et al, 2016[126]	To investigate if coercion is involved in decision-making of medical undergraduate and postgraduate students participating in research; Mumbai, Maharashtra, India	Questionnaire survey; n=300
**B. Secondary Research: Reviews of documents**		
Bavdekar, 2009[64]	To determine the extent to which issues related to the provision of free treatment and compensation for research-related injury are addressed in the informed consent documents from protocols submitted to ethics committees; Mumbai, Maharashtra, India	Documentary analysis; n=138
Bhide et al, 2016[66]	To evaluate the impact of the 2013 regulatory changes on ethics committee structure, review process, outcomes and administration; Mumbai, Maharashtra, India	Documentary analysis
Chaturvedi et al, 2017[139]	To assess if clinical trials were in line with the health care needs of the country by auditing the clinical trials registry of India	Database analysis (Clinical Trials Registry of India)
Jadhav et al, 2015[85]	To evaluate completeness of ethics application forms submitted for review to ethics committees; Maharashtra, India	Documentary analysis; n=100
Jhanwar et al, 2010[86]	To assess the ease of readability of translated informed consent forms used in psychiatric clinical trials; Varanasi, Uttar Pradesh, India	Documentary analysis; n=30
Kundapura et al, 2013[95]	To assess compliance of informed consent documents with regulations; Pune, Maharashtra, India	Documentary analysis; n=50
Kuyare et al, 2014[96]	To assess queries raised by ethics committees in uninitiated studies and whether these studies obtained ethics approval elsewhere; Mumbai, Maharashtra, India	Documentary and database analysis; n=219 uninitiated studies (minutes of ethics committee meetings) and Clinical Trials Registry-India data
Padhy et al, 2011[107]	To assess compliance of informed consent documents from protocols submitted to ethics committees in relation to the Indian Good Clinical Practice guidelines; New Delhi, India	Documentary analysis; n=300

Patwardhan et al, 2014[110]	To compare the quality and completeness of data and documentation between an investigator-initiated trial and an industry-sponsored study; Mumbai, Maharashtra, India	Documentary analysis and data from 42 patients (28 from investigator-initiated trial; 14 from industry-sponsored study)
Selvarajan et al, 2013[141]	To evaluate the trends in clinical trials in India compared to other countries, and make comparisons to India's disease burden	Database analysis (multiple clinical trial registries)
Shah et al, 2016[118]	To check completeness and find errors in application forms submitted to ethics committees; Bhavnagar, Gujarat, India	Documentary analysis; n=100
Shetty et al, 2012[120]	To review ethics committee application forms for completeness; Mumbai, Maharashtra, India	Documentary analysis; n=445
Shetty et al, 2012[119]	To monitor adherence to protocol and the informed consent process through clinical research site visits by ethics committee members; Mumbai, Maharashtra	Documentary analysis; n=7 site monitoring reports
Marathe et al, 2018[99]	To study the payments for participation allowed by ethics committees and reasons for payments; Mumbai, Maharashtra, India	Documentary analysis; n=227 studies (ethics application forms, protocols, informed consent documents, correspondence of ethics committees with investigators)
Nishandar et al, 2019[106]	To evaluate status of registered, re-registered and accredited ethics committees in India in relation to regulations; India	Database analysis (Central Drugs Standard Control Organization, National Accreditation Board for Hospitals and Healthcare Providers, Clinical Trials Registry of India and Census data); n=1268 ethics committees
Taur et al, 2011[123]	To determine extent to which ethics committees comply with requirements mentioned in guidelines and regulations while issuing letters of approval; Mumbai, Maharashtra, India	Documentary analysis; n=20 (approval letters from 20 ethics committees)
**B. Secondary Research: Journal articles**		
Bavdekar et al, 2008[63]	To determine proportion of research manuscripts reporting on ethical clearance and obtaining informed consent and/or assent in two paediatric journals published from India	Documentary analysis; n=132 manuscripts
Chaturvedi et al, 2009[71]	To examine whether informed consent and ethical approval were reported in published psychiatric research in one psychiatric journal published from India	Documentary analysis; n=157 manuscripts
Chin et al, 2011[74]	To explore how often journal articles reporting HIV research sponsored by a developed country but conducted in a developing country mention ethics approval from both countries; four countries including India	Documentary analysis; n=50 manuscripts from India
Klitzman et al, 2010[93]	To explore how often human subject research on HIV reported a funding source and conflict of interest in four countries, including India	Documentary analysis; n=79 manuscripts from India
Klitzman et al, 2011[94]	To investigate how often human subject research on HIV reported on ethical approval in four countries, including India	Documentary analysis; n=79 manuscripts from India
Tharyan et al, 2013[124]	To evaluate improvements in Indian journals' editorial policies and reporting quality of RCTs and to compare with reporting quality of protocols in the Clinical Trials Registry-India	Documentary analysis; n=67 Indian medical journals; 145 published trial reports; 768 randomised trials registered on the Clinical Trials Registry-India
