# Supplementary material for: What empirical research has been undertaken on the ethics of clinical research in India? A systematic scoping review and narrative synthesis
Source: BMJ Glob Health. 2021 May 18;6(5):e004729. doi: 10.1136/bmjgh-2020-004729 (PMC8137180; doi:10.1136/bmjgh-2020-004729)
Supplement: Supplementary data [file bmjgh-2020-004729supp007.pdf]

Supplementary file 7: Full report of narrative synthesis

A.1. Comprehension of the clinical trial/research informed consent form and verbal information provision in a real or hypothetical research study: Lay (and some professional) participants

Number of studies tagged to topic: 10[62,65,78,80,82,87,91,104,116,117]

Methodological aspects and limitations:

- Comprehension was assessed through questionnaire surveys conducted with sample sizes that were generally small (n $\leq$ 50)[62,65,78,80] to moderate (n = 100 to $\leq$ 200),[82,91,104,116] with two larger studies[87,117] (n = 1334 and 368). Most studies, including small-scale, carried out inferential statistics (9/10).
- Most studies were within a single centre. [62,65,78,80,82,91,104] Some did not mention the time period between information provision and comprehension assessment[62,87], with a few conducted more than a year[78,104] to four years[117] after the informed consent interaction.
- Response categories in tools were not clear/not provided in some studies[78,104,116,117], and in others they ranged from multiple-choice questions[62,65] to combinations of categorical and open-ended questions[82,87,91].
- Method of questionnaire administration (6/10), source(s) for questionnaire content (6/10), and whether questionnaire was piloted/validated (5/10) were sometimes not clear or provided.
- Demographic information such as age, gender and education and/or literacy levels were more often provided (although not always) than religion and indicators of socio-economic status such as employment and income.

Synthesised findings:

- Lay group studies (n=8): Participants were reported as comprising a majority of those educated to primary level or more,[65,117] secondary level or higher,[80,82] not completing secondary level[116] or as mostly literate.[78,87] Some studies suggested that lay participants (and/or their relatives) mostly had difficulty understanding or recalling information on the study background,[62] what is a clinical trial,[82] study treatment being unproven yet as the best for their condition,[82] the condition under study,[117] risks[80,87] and benefits[78]. Additionally, in RCTs, randomisation,[65,116] blinding[65] and the need for a placebo[65] appeared difficult to comprehend. A few studies reported that more participants were found to understand that they were taking part in a research study,[87,116] study procedures (e.g. blood samples),[87,116] and confidentiality.[78,87] Study purpose was reported as both well[78] and poorly understood.[116,117] Comprehension on different aspects of autonomy appeared to vary. Some studies indicated that while most participants understood the voluntary nature of participation,[87,116,117] a nuanced understanding of their rights may be lacking as they did not appear to be aware that they were free to withdraw at any point[116,117] or that declining participation would not adversely affect their or their children's regular medical care.[116] In contrast, the rights of participants[62,78,82,87] (such as alternatives to taking part, access to standard care, declining participation or withdrawing)[82,87] were reported to be well understood in some studies.
- Some studies reported that there was no statistically significant variation in comprehension by age,[78,82,87] gender[78,82,87] (except for risk-related information which was better understood by women)[87] socio-economic status,[78,117] income,[65] employment status[87] and time taken for consent.[78] There was variation in comprehension by literacy reported in a large study[87] and no variation in a smaller study.[78] Similarly, there was variation in comprehension by education in a large study (maternal education)[117] and no variation in small to moderate-sized studies.[62,65,82] One study reported no difference in comprehension between patients who were illiterate and those who were educated (non-college or college).[82]
- One RCT that compared group and individual counselling for informed consent did not find a difference in comprehension of key elements between the two groups[116] and an observational study that compared informed consent comprehension scores before and after the introduction of the mandatory AV recording for the consent process found better comprehension after[78] (the duration between consent and questionnaire administration was shorter in the AV group). One study reported that comprehension was significantly higher in pharmaceutical industry-sponsored trials compared to investigator-initiated trials (which the authors have attributed to the elaborate informed consent forms and the lengthier informed consent process in the former).[82] In some studies, the comprehension assessment was used to provide further information to participants on the topics in which they had a lower score, [62,87] with one incorporating a cut-off of comprehension scores $\geq$ 80% to be eligible for study enrolment (unclear if participants were retested after further information provision). Studies did not explore what may constitute optimal understanding or information provision.
- Professional group studies (n=2): Studies that assessed comprehension in a real cohort study amongst nursing students (undergraduate and postgraduate)[104] and a hypothetical clinical trial amongst medical students (undergraduate),[91] both reported insufficient levels of understanding, although these scores appeared much higher than the scores reported for the lay groups.

Paramasivan S, *et al. BMJ Global Health* 2021; 6:e004729. doi: 10.1136/bmjgh-2020-004729

A.2. Knowledge of and attitudes/perceptions to clinical trials/research more generally (not in the context of specific studies):

i. Lay participants

Number of studies tagged to topic: 7[69,88,113,122,127,134,140]

Methodological aspects and limitations:

- Amongst the four questionnaire surveys, the larger study (n=400)[122] used descriptive statistics and the more moderate-sized studies (n ~ 175 to 240)[69,113,134] used inferential statistics.
- Two studies administered the same 20-item questionnaire, with Yes/No and True/False/Not Aware response categories to elicit attitudes/beliefs[69,122] that were also reported as knowledge or awareness.
- Aspects such as method of questionnaire administration[122], validation/piloting[134] and source(s) that informed the content of the questionnaire[113] were not mentioned in some studies.
- Demographic information such as age, gender and educational qualifications of participants were generally provided.
- Two were qualitative studies (n=24 and 14)[88,127] that used interviews and/or focus groups, but one study reported the findings descriptively with numerical presentation of results (without an interpretive account).[88] Findings from another qualitative study, where knowledge exploration was not the focus, have also been included here (group discussion with 50 healthy volunteers; other findings from this study have been included in A5 and A6).[140]

Synthesised findings:

- More than half the participants in three of the questionnaire survey studies were educated to graduate level or more[69,122,134] and in the fourth, the majority of participants (72%) had had more than 7 years of education (i.e. at least primary level).[113] Four studies accessed participants primarily from hospital settings[88,113,122,134] and one from public locations.[69] Qualitative studies mostly comprised participants educated to above primary school level[127] and to graduate/post-graduate level.[88]
- Knowledge: Qualitative studies reported that participants (including graduates) who were taking part in clinical research (bioavailability/bioequivalent studies)[140] and those who had not previously taken part in CTs[88] were unaware of what they were or involved (including study name/purpose; only aware that blood would be drawn from them, they may develop rashes or a headache and that they should report other symptoms), that non-English speakers had not heard of the word 'research' and were not familiar with the local translations for the word[127] and that lay participants were generally unaware of the rules and regulations of biomedical research or the role of ethics committees in protecting patient interests.[127] The proportion of participants who said they had heard of clinical trials or clinical research varied considerably across the questionnaire surveys from ~25%[69,134] to 60%.[113] On exploring what participants knew, some studies reported that knowledge was basic[88,113] (i.e. associating clinical trials/research with finding something new) to incorrect among some participants.[134] Those who had heard of 'research' appeared to have positive expectations of it.[127]
- Attitudes: Studies reported that lay participants had overall positive attitudes towards clinical research (i.e. that it benefits community, society, humanity[69,113,122] and is an important step in developing new treatments and advancing medical science[69,122,127,134]), with some noting that participants' main areas of concern were around the protection of participant confidentiality, compensation for participation and adverse outcomes,[69,122] unethical practices in trial conduct[127] (such as fudging data, profiteering, using people as guinea pigs), and lack of trust in pharmaceutical research.[134] Research by academic institutions appeared to be more trusted than those by pharmaceutical companies, with just over half the participants in some studies trusting the government to protect the public against unethical research.[69,122]

ii. Professional participants

Number of studies tagged to topic: 5[75,100,112,129,131]

Methodological aspects and limitations:

- Amongst the four questionnaire surveys, two studies (n=133 and 395) used inferential statistics[75,131] and two (n=102 and 257) used descriptive statistics.[100,129]
- Two studies used the same questionnaire, but the source(s) used to inform questionnaire content was unclear.[100,129] Studies had some explanation of validation/piloting of questionnaires used, but some did not explain questionnaire administration clearly.[75,100,129]
- Demographic information such as education were usually provided but not gender and age of participants in some instances.[100,129]
- Other key information of relevance, i.e., prior clinical trial/research training (curricular or extra-curricular) and experience was usually provided.
- One qualitative study employed in-depth interviews (n=50).[112]

Synthesised findings:

- Studies were conducted with doctors,[75] healthcare students (pharmacology[100] and medicine[129]), a combination of medical students and teachers[131] and Frontline Health Service Providers[112] (FHSPs providing services to female sex workers and men who have sex with men; includes doctors, nurses, counsellors, outreach workers, peer educators, programme managers).
- Knowledge: Studies reported basic level of knowledge of clinical trials/research amongst doctors [75] and medical students and teachers,[131] but lack of familiarity with methodological aspects and regulatory requirements of clinical trials.[131] Knowledge on aspects such as patient confidentiality and rights (e.g. to withdraw after study enrolment and for compensation due to study related injury) appeared adequate[75], while knowledge on aspects such as guidelines, regulations and regulatory authorities appeared inadequate.[75,100,129] The qualitative study with FHSPs found that more than half the participants across different educational backgrounds had little or no awareness of what a clinical trial entailed.[112] Participants were unfamiliar with the English term 'clinical trial' as well as the local translation of the term (similar to lay participants above). Those who had some knowledge of the existence of clinical trials (usually participants with degree level education), admitted to having limited knowledge and some confused clinical trials with routine medical tests and procedures.[112]
- Attitudes: Some studies reported overall positive attitudes to clinical trials/research amongst doctors and medical students/teachers[75,131] (such as 'clinical research is important for the progression of medical science'). Negative attitudes towards pharma or industry-sponsored studies were reported[75] (e.g. majority believed that clinical trials carried out for academic purposes were more ethical/scientific than industry-sponsored trials, patients were exploited and legislations were inadequate in industry-sponsored trials). Clinical trials conducted in India were not considered of good quality by many[131] and there was support for including clinical trials in detail in the undergraduate/postgraduate medical curriculum.[75,131]

A.3. Knowledge, attitudes/perceptions and practices in relation to research ethics (including informed consent): Professional (and some lay) participants

Number of studies tagged to topic: 16[67,77,81,98,101,103,109,126,127,132,133,135-138]

Methodological aspects and limitations:

- Of the 12 questionnaire surveys, seven (n=81, 100, 114, 172, 213, 300, 385) stated they used inferential statistics[77,81,109,126,132,135,137] (but one was reported entirely descriptively;[109] the same dataset was used in another article to report on ethics committees and has been included in section E). Five surveys, including a large one (n=29, 942, 34, 181, 100) used descriptive statistics[67,98,133,136,138] (the findings from the large survey have been minimally used in the synthesis due to discrepancies across numbers/proportions mentioned in tables, results and discussion).[98]
- Details that were unclear/not provided included questionnaire administration,[67,126,132,138] source(s) used to inform questionnaire development[67,98,109,132,133,135-137] and validation/piloting.[109,132,133,136,137] Three studies used similar questionnaires.[81,98,137] It was not always clear if knowledge on the topic was self-reported or objectively assessed.[98,135] Generally, attitudes were better explored and reported on than knowledge.
- Demographic information such as education, age and gender were usually provided (all[133] or some aspects, i.e. age and/or gender, were sometimes not provided[67,109,135,136]).
- In some instances, information was unclear/not provided on whether participants had had prior clinical trial/research training[67,77,98,126,133,135-138] and experience[77,98,133,136].
- One study was reported as a mixed methods study; the reporting of methods and results were however not clear, including the qualitative aspects, so findings have been used minimally in the synthesis.[132] In another study, some questions were not framed clearly and some knowledge questions appeared to assess attitudes[138]. One study was authored by employees of a pharmaceutical company[67] and two studies by employees of clinical research organisations (CROs).[133,136]
- Two qualitative studies employed interviews, one with professional group participants (n=19)[101] and another[103] with both lay (n=32) and professional participants (numbers not available). Lay participant views from this study[103] as well as from another qualitative study[127] (methods included in section A2.i.) have also been included in this section on professional participants as it covers similar themes.

Synthesised findings:

- Four studies were conducted with dental professionals (dental faculty only[81,98] or with dental students and faculty[77,137]); one was with medical professionals (medical students and faculty);[135] and three were with professionals primarily from clinical research organisations/sponsors, but also comprising other stakeholders such as investigators and ethics committee members (henceforth referred to as clinical research professionals for simplicity).[67,133,136] Of the four studies that focused on informed consent, one was with clinical research

professionals[109] and three were conducted with medical students.[126,132,138] One qualitative study explored ethics committee members' views on issues such as ethical guidelines.[101] A further two qualitative studies were primarily with lay participants - focused on clinical trial participants' experiences of participation[103] and on biobanking and biomedical research in general[127] (the only two lay participant views included in this section).

- Knowledge:
  - *Research ethics:* Studies reported that there appeared to be gaps in self-reported knowledge (i.e. where participants where asked if they were familiar/aware of a particular topic or not) of research ethics[81,137] or poor actual knowledge[77] (i.e. when reported as tested/assessed, although unclear what questions were asked) amongst dental professionals.
  - *Ethical guidelines:* Self-reported knowledge on national/international guidelines for research ethics was noted to be poor among dental[98] and medical professionals.[135] Ethics committee members (from 11 committees) in a qualitative study were generally found to be aware of national ethical guidelines (but not international).[101]
  - *Informed consent:* One study reported that knowledge of informed consent was good as all participants (medical students) knew that informed consent: was not only verbal consent, should include information that it is a research study, includes patient autonomy to withdraw at any time, is mandatory in prospective studies and should not be obtained with undue inducement. Most medical students were also noted as being aware that informed consent includes aspects such as study duration, information on risks/benefits of participation, statements on confidentiality/privacy and is mandatory in observational surveys.[138] In general, studies reported that medical students had good knowledge of informed consent but poor attitudes and practices in relation to the same.[132,138] In a qualitative study with clinical trial participants (lay group), some appeared confused between the signing of the informed consent form and filling of the questionnaire for the trial.[103] In another qualitative study with lay participants, to most respondents, 'to consent' meant 'to agree' and that this was done by signing (however, this was in general seen as providing protection to the doctor/researcher/hospital than the patients).[127]
- Attitudes:
  - *Research conduct:* There appeared to be some support, amongst dental professionals, for fabricating data to improve research outcomes if it did not harm patients (ranging from 12% to 44%).[81,98,137] A fifth of resident doctors appeared willing to undertake research that was rejected by ethics committee.[135] There was all round support for the need to protect confidentiality of participant data and to take measures to prevent accidental exposure of patient data.[81,98,137]
  - *Informed consent:* Some ethics committee members felt that the informed consent form was merely a tool to obtain signatures, it was often not read to patients and not in local languages.[101] Similarly, more than half the clinical research professionals felt that the informed consent process does not truly inform patients and is focused on legal compliance.[67] More than 90% of dental professionals were in favour of informing patients of the risks and benefits of research,[81,98,137] always including the patient's signature as part of informed written consent, seeking informed consent when involving patients with invasive procedures and for the use of biological samples (but lesser support in relation to blood samples, 78% and 44%).[98,137] Most post-graduate medical students ($\geq$ 80%) believed that informed consent should be explained in the local language, be obtained before the start of research work and patients should be allowed to withdraw after signing informed consent; fewer (66%) believed that a witness was absolutely necessary during informed consent.[138] Majority (> 80%) of clinical research professionals believed that participants were offered the opportunity to ask questions, were able to refuse participation[133] and had full understanding that there was no compulsion to participate,[67] although very few from the same group believed that patients were truly autonomous.[67] Most clinical research professionals in another study believed participants did not have full understanding that there was no compulsion to participate[109]. More than half of clinical research professionals believed participant rights and alternative treatment options were explained during the informed consent process and about half felt patients were adequately informed about trial participation and informed of risks,[133] but most clinical research professionals in another study believed participants were not properly informed of risks.[109] Many clinical research professionals believed that the informed consent process should be monitored by ethics committees or patient research advocates.[109]
  - *Informed consent forms:* Most clinical research professionals had concerns about information on funding on informed consent forms, along with information provided on study purpose, possible risks/benefits and right to withdraw; they believed forms should be simplified and include pictorial images.[109]
  - *Research ethics education:* Most dental professionals were in support of research ethics education for postgraduate students, investigators and ethics committee members.[81,98,137]
  - *Clinical trial drug industry:* Most clinical research professionals believed that the industry in India is growing, but that India is not utilising its full potential and delays in regulatory approvals were a key hurdle to the growth of the clinical trial industry in India (lack of trained investigators/site staff, unethical practices and public awareness also selected as hurdles by many, but lack of patient population and increasing costs of clinical research in India were not).[136]

- Practices: One study reported 'unsatisfactory' behaviour in relation to how frequently dental professionals used scientific journals/internet regarding research ethics, whether they maintained accurate patient records for research and whether they attended training programmes in research ethics.[77]
  - *Informed consent:* Majority of medical professionals had obtained written informed consent during research.[135,138] Proportion who did this in the local language varied (≤ 50%[135] to > 80%[138]). The majority (> 80%) of medical professionals in one study stated that they obtained the signature of an impartial witness alongside that of participants' on the consent form, handed over the participant sheet while obtaining informed consent and explained to participants that they were in a research study[138]. In another, only a third of medical professionals reported taking consent in the format advocated in national guidelines[135] and far fewer (~ 12%) provided a written copy of the written consent to patients.[135] There was no research on what information recruiters usually discuss in an informed consent interaction or what patients expect to be informed about.
  - *Coercion:* In one study that explored if medical students felt coerced into research participation, a quarter of participants stated they had participated in research study/studies due to faculty requests, a few did not know they could refuse participation, a third disagreed that participation was entirely their own choice and two-thirds said they had participated despite not wanting to. Majority also felt that faculty would like it if they participated and that it will help their academic grades. Overall, authors concluded that medical students felt under pressure to participate in research studies and were concerned about the repercussions of refusal.[126]
  - *Experiences of informed consent process (lay participants):* Some clinical trial participants in a qualitative study stated that they were not given detailed (or sometimes any) information about the trial before enrolment and that the benefits of the drug being tested were sometimes emphasised and presented as the best option available. Many said they had signed the consent form without understanding the contents as they trusted their doctor. All participants appeared to be aware of their right to withdraw, but their accounts indicated that their decision making for participation may not have been truly autonomous and voluntary (mediated by factors such as gender norms).[103]

A.4. Knowledge, attitudes/perceptions and practices in relation to Ethics Committees (including composition, functioning, performance, capacity, review process): Professional (and some lay) participants

Number of studies tagged to topic: 18[67,68,73,76,81,89,92,97,98,101,102,108,121,127,130,133,135,137]

Methodological aspects and limitations:

- Of the seven questionnaire surveys, three used descriptive statistics (n=25, 73, 29)[76,89,102] and four used inferential (n= 52, 180,385, 30),[68,97,108,130] of which one was reported entirely descriptively.[108]
- Some information was usually provided on questionnaire administration, with information being limited[97,102,130] or unclear/not provided[68,76,108] on validation/piloting; and unclear/not provided on source(s) used to inform questionnaire development.[76,97,108,130]
- Demographic information was not always provided on education[76,130], gender[76,89,97,108,130] and age[76,97,102,130] of participants.
- Information was sometimes not provided on clinical trial/research training[76,89] and clinical trial/research experience.[76,97,102,130]
- Three qualitative research studies employed interviews (n=6, 17, 14).[73,92,121]
- (Note: Methodological aspects of seven studies included in the synthesis here are within the previous section, A3[67,81,98,101,133,135,137] Also, findings from one study with lay participants that briefly explored views on ethics committees is included here[127]).

Synthesised findings:

- Questionnaire surveys were conducted with dental professionals (dental faculty only[81,98] or with dental students and faculty[137]); medical professionals (medical students and faculty[135] or medical faculty only[97]); ethics committee members,[68,76,102,130] and clinical research professionals (comprising investigators from contract research organisations/public hospitals with/without ethics committee members and sponsors).[67,89,108,133] Four qualitative studies were with ethics committee members[73,92,101,121] and one was with the general public.[127]
- Knowledge: *Ethics committee composition and functioning:* Self-reported awareness (i.e. where participants where asked if they were familiar/aware of a particular topic or not) of the functions of ethics committees amongst dental professionals[81,137] and self-reported awareness of the composition of ethics committees amongst medical professionals[135] were both reported to be limited. Majority (56%) of medical faculty in one study were reported to have below-average to average actual knowledge (i.e. when reported as tested/assessed) on ethics committee composition and functioning (explored quorum requirements, member composition, lay representation, frequency of meetings, submission deadlines).[97] In another study, ethics committee members were reported to be aware of the requirement of a quorum to conduct a meeting, but not how many members constituted a quorum.[102] Some

studies asked whether participants knew of the presence of any ethics committees and reported that the majority did.[81,135] Lay participants in a qualitative study were reported as unaware of the role of ethics committees in protecting the interests of research participants or in addressing the violations of their rights.[127]

- Attitudes:
  - *Ethics committee functioning:* There was widespread support for the existence and need for ethics committees among dental professionals,[81,98,137] but limited satisfaction amongst medical professionals and clinical research professionals regarding ethics committee functioning in some studies[108,135] and high levels of satisfaction in others (with clinical research professionals).[67] About half the clinical research professionals scored ethics committees 5 or over (on a scale of 1 to 10) in relation to their independence[133]. Conflict of interest was considered a key reason for committees' lack of independence[133] and as a barrier to committees' functioning by investigators.[108] Pressures from senior management was also considered a reason for committees' lack of independence by clinical research professionals in one study,[133] but only few felt that pressure from sponsors was a barrier faced by ethics committees in another study[108]. Majority of ethics committee members felt that the committees' functions should include mediating between the media and researchers, monitoring serious adverse events, ensuring the community benefits from the research,[68] protecting patient confidentiality and imparting research ethics education to investigators.[101] Most clinical research professionals believed that auditing ethics committee performance by third parties and the registration of ethics committees will improve the functioning of ethics committees and ethical standards.[108]
  - *Regulatory changes:* Ethics committee members discussed the evolution of stricter guidelines for how ethics committees function and felt that the 'bar has risen' over time.[121] In another study with ethics committee members from 25 committees, members were in support of regulatory changes (of August 2016, where ethics committees have to take responsibility for decisions such as the number of trials per investigator), but felt that the changes were too many, too often and a burden to committees.[76] While most ethics committee members stated feeling empowered to take a decision on approving the number of studies per investigator,[76] fewer investigators were in favour of restrictions on trial numbers allowed per investigator.[89] Ethics committee members felt that there was lack of clarity on the role of independent ethics committees.[76] *Accreditation/registration:* Most ethics committee members were in favour of accreditation for all committees, but identified challenges they encountered with the process for renewal of registration of ethics committees (such as lack of clarity with requirements and cumbersome documentation, lack of institutional support and resource constraints and lack of acknowledgement after submission of documents). [76]
  - *Ethics committee composition:* Most ethics committee members felt that the committees should be reconstituted every two years and that those invited as members should be experts in their field and trained in ethics.[68]
  - *Ethics review:* In general, majority of dental professionals supported the need for ethical review for all human research,[81,98,137] except surveys and retrospective studies (this was amongst dental and medical professionals).[98,135] In the same vein of support for ethical reviews, majority of dental professionals disagreed that ethics approval was not necessary due to the presence of scientific committees[81,98], ethics approval delays research and makes it harder for researchers[81,98,137] and ethics review should be restricted to international collaborative research.[81,98,137] Two-thirds of ethics committee members (of 25 committees) believed that the scientific review committees and the ethics committees should be kept separate.[76] Most, but not all, ethics committee members disagreed with trials starting before ethical approval to save time.[102] More than half the clinical research professionals did not feel that the safety review by ethics committees was adequate.[133] Most clinical research professionals felt the ethics review process benefits research, but also that ethics committees failed to understand research protocols/methodology and sometimes over or under estimated risks of clinical trials.[108] The notion of 'ethics committee shopping' was discussed by committee members, where investigators/sponsors went elsewhere if refused approval at the first.[121] There was overwhelming support for a single national research ethics committee to consider multicentric trials amongst clinical research professionals,[89,108] which is likely to help prevent 'ethics committee shopping', but there was less support for this amongst ethics committee members.[76]
  - *Research ethics/GCP training:* Majority of ethics committee members[68,92] and clinical research professionals[133] were in support of research ethics/GCP training for ethics committee members (although fewer, i.e. ~ 40%, ethics committee members were in support of this in another study[102]). Clinical research professionals also supported wider training for ethics committee members, for example, in regulations and roles/responsibilities of each member.[108] Some ethics committee members stated that training was challenging to organise for doctors who were busy and that doctors may not require intensive ethics training as they were already sensitised to patient issues and aware of the role of ethics in clinical research.[92]

- *Ongoing monitoring and on-site visits:* Ethics committee members from 11 committees in one study were in support of ongoing monitoring of trials by ethics committees,[102] while in another study members from five committees were not in support of this and believed monitoring should be the responsibility of third parties or sponsors/investigators.[92] Most clinical research professionals believed that improving the review process through on-site visits will contribute towards improving the functioning of ethics committees.[108]
- *Guidelines/regulations:* One qualitative research study suggested that ethics committee members intense focus on informed consent, guidelines, legality and regulations in their accounts may be because this was a way for committees to gain credibility amongst researchers; strict proceduralism was felt to overtake protection of participants' interests and being humanistic.[121] Another qualitative study provided an example where institutional bias was observed (the use of a placebo in a trial was discussed in relation to protecting the institute's interests rather than as a moral dilemma) and that participant protection was often the by-product of the need to safeguard an institution's legal accountability.[92]

- Practice:
  - *Ethics committee functioning and composition:* Members from eleven ethics committees reported that they function independently and with appropriate representation of people with different qualifications as stipulated by national guidelines.[102] Most ethics committee members scored themselves above 5 (one a scale of 1 to 10) in relation to their involvement in meetings and those from non-medical backgrounds mostly stated that they did not feel restricted by their background while participating in meetings.[130] Most ethics committee members (from five committees) noted that there was an arbitrariness in member selection, with no policies on selection and reliance on informal networks, especially for members not affiliated to the institutions in which the committee is based. Members affiliated to the institutions, on the other hand, appeared to have limited choice in refusing membership in a committee[92]. Two ethics committees scored 62% and 67% on a quality assurance self-assessment tool for ethics committees.[73]
  - *Ongoing monitoring and on-site visits:* Nearly all ethics committee members from 11 committees said they undertook periodic ethics reviews of ongoing trials, but far fewer said on-site monitoring was conducted.[102] In another study members from five ethics committees said they did not undertake monitoring of ongoing trials.[92] Nearly two-thirds of committee members (of 25 committees) said they did not have a well-devised plan to visit sites for monitoring during study conduct and just over a third stated that their committees had visited sites for monitoring ongoing studies.[76] Ethics committee members from two committees in another study said they required annual and end of study reports from investigators.[73] Members from 11 committees in a qualitative study highlighted variations in practice.[101] Some (contrary to national guidelines) did not see committees having a role in ongoing monitoring of research conduct and management of information and explained that their role was over once approval was granted. Others stated they intermittently investigated whether studies were carried out appropriately.[101]
  - *Workload and working patterns:* The increasing workload of ethics committees was frequently discussed across studies, including that limiting the total number of trials handled by committees will improve its functioning.[108] Some reported the frequency of meetings across ethics committees (once/week to once/two months[102] or 25-70 per year[73]), the number of protocols reviewed per meeting (1 to 20 per meeting;[102] 50 protocols once/month in ethics committees of public hospitals versus 2-6 protocols once/month in those of private hospitals)[92] or the number of meetings attended per year by members (1 to 10 per year).[130] Some public hospital committees appeared to combine the scientific and ethical review at their meetings and were reported as having lesser administrative support than those in private hospitals.[92] Most members from 11 committees stated that they received proposals two weeks in advance of the review meeting, results were communicated to investigators within a week and that all documents were archived for five years.[102]
  - *Ethical review:* One study raised in detail the dilemmas faced by ethics committees in India (as well as Sri Lanka and Nepal) in relation to the growth of pharma industry (pharmaceuticalisation) and the ethical review process. Committees face increasing the pressure to assimilate within the international standards of ethical review, while also being cognisant of their larger responsibilities towards protecting not just research participants but also national interests[121] (for instance, in ensuring research does not reinforce existing health and social inequities).
  - *Honorarium:* Most ethics committee members (from 11 committees) said they received an honorarium for their time.[102]
  - *Documents reviewed:* Amongst two ethics committees studied, both were reported as having a policy for how protocols were reviewed, when members received the protocol and supporting materials for review, but only one had a checklist for documenting their ethical assessment.[73]
  - *Aspects reviewed:* Some committee members outlined the privacy/confidentiality[73,101] and informed consent[73] aspects that were considered by investigators and reviewed by ethics committees (i.e. how data collected was protected, whether lock and key or electronic; process/setting of obtaining informed consent; reading level of informed consent forms; and whether they covered the basic elements of informed consent).[73,101]

- *Guidelines followed:* There appeared to be variations in how and which ethical guidelines were followed by ethics committees.[101] All ethics committee members (from 11 committees) stated they followed national guidelines (ICMR), but fewer mentioned international guidelines (e.g. ICH-GCP, WHO GCP).[102]
- *Training:* Ethics committee members recognised that they had high training needs and majority of members (from 25 committees) said that their committee has a training plan and members are trained when there are new regulations.[76]

A. Primary research: Perceptions, experiences, practices/processes

A.5. Informed consent processes: Lay (and some professional) participants

Number of studies tagged to topic: 13[70,72,79,83,111,140] including findings from seven studies[76,78,89,103,109,127,133] where the focus was not on informed consent

Methodological aspects and limitations:

- One large (n=4382)[111] quantitative study was based on observations of informed consent discussions and the other was a questionnaire survey (n=150)[72], with both employing inferential statistics.
- Of the three qualitative studies, one employed interviews (n=8) and observations of consent interactions (n=5)[79], another involved audio-recordings of consultation recordings (n=100)[83] and the third was a case study of a contract research organisation (CRO) conducting bioavailability/bioequivalent (BA/BE) studies, comprising interviews with CRO staff (n=8), group discussion with healthy volunteers (n=50) and observations of informed consent discussions (n=40).[140]
- A mixed methods study involved a questionnaire survey (n=332; descriptive) and one focus group discussion.[70]
- Methodological aspects of the additional seven studies[76,78,89,103,109,127,133] are in other sections.

Synthesised findings:

- Studies were mainly conducted with lay participants (general public,[72] healthy volunteers in BA/BE studies,[140] potential clinical trial participants,[70,79] including parents of children[83,111]). Three studies also included views of researchers[70,79] and CRO staff.[140]
- Only one study described the process of customising the informed consent process to the trial population. The informed consent procedure for an RCT with people with schizophrenia was developed with prior feedback from participants/caregivers, incorporated the feedback received (such as simplifying the information sheets, developing a flip chart with diagrams to explain key study elements, making the consent procedure more interactive) and then evaluated the feasibility of this informed consent process from multiple perspectives. The informed consent process and the use of the flip chart were found to be useful by participants and study personnel. Study personnel found the manual-based training and ongoing support to be helpful and noted that concepts such as trial, research and randomisation were difficult to convey and required considerable time to explain.[70]
- <u>Patient participation in informed consent discussions:</u> The questions asked by parents/guardians of potential child (infant) participants during discussions varied from 13% in a study where the discussions where preceded by a community information session (study physicians/research nurses were not involved in consent process; study personnel trained on ICH-GCP guidelines were instructed to encourage questions from participants and note down questions/comments at the back of the consent form when questions went beyond simple clarifications of informed consent form; study was conducted by an organisation that has provided charitable health services in the community for more than 30 years)[111] to 55% in audio-recordings of consultations.[83] Most frequent questions asked include who to contact in an emergency, risks to child, questions specific to the condition being studied (such as tuberculosis) and benefits to child/family of participants.[83,111] Education,[83,111] higher socio-economic status, and the presence of both parents were associated with asking questions.[111] Some participants in a qualitative study (interviews exploring hypothetical trial participation), especially those who were less educated and did not know the meaning of research, stated that they would not ask the doctor any questions about the trial, despite lacking sufficient information.[127] In a qualitative study of healthy volunteers for BA/BE studies, observations of informed consent discussions revealed that the volunteers' concerns revolved mainly around the payment they would receive for participation than about their own health.[140]
- <u>Recruitment process/experience and informed consent process:</u> A qualitative study that examined a CRO as a case study found that healthy volunteers were recruited for bioavailability/bioequivalent studies through lists created through networks and middlemen who are paid a commission for recruitment. These volunteers constituted a pool of readily-

available participants regularly approached for participation, with many volunteers exceeding the maximum number of studies they are allowed to participate in per year. CRO staff stated that some CROs have systems in place to thwart such irregularities, but others did not, facilitating serial participation. CRO staff also noted that most volunteers had decided to participate much before they attended the informed consent discussion or saw the consent documents, with the subsequent informed consent process being a mere formality. Contrary to accounts of family-based models of informed consent being the norm, volunteers were unaccompanied during discussions and nearly all (48/50) said they decided to participate in the bioavailability/bioequivalent studies without informing their families as they would not allow the volunteers to participate and would see the volunteers as selling their bodies for money.[140]

- Audio-visual (AV) recording of informed consent discussions:
  - *Acceptance:* In a study that used a hypothetical scenario to assess acceptability, a third of the (lay) participants refused consent for AV recording of consent,[72] whereas nearly all (lay) participants who expressed an initial willingness to participate in a real vaccine trial agreed to undergo AV consenting process.[83] In a study where AV recording process was observed, it was noted that patients and investigators were uncomfortable (self-conscious) due to the process,[79] whereas authors in another study noted that while patients seemed intimidated by the AV consent process at the beginning, they became more relaxed and comfortable after it was explained and they started to participate in it.[83]
  - *AV process:* Consent discussions that were audio-recorded were described as being undertaken in private spaces[79,83], without any other individuals present[79] or with an impartial witness if the patient was illiterate,[83] after separate consent for AV recording,[79,83] with recordings stored with password protection.[79] Time taken for the AV process varied from 30-45 minutes[83] to an hour-and-a-half to two hours.[79]
  - *Perceptions of AV recording:* Support for the AV recording process among professionals varied (nearly two-thirds of clinical research professionals,[133] just over a third of investigators[89] and investigators in general in a qualitative study[79] were reported to be in favour of the AV recording of informed consent). Investigators expressed concerns about the lack of guidance and training to support them[79] and investigators and patients were concerned about the extra time that was required to undertake the AV consent process.[79,89] Key informants (investigators, from sponsor/contract research organisations, ethics committee members) and patients had privacy and confidentiality concerns with the process.[79,89,103] Other concerns included that it may cause anxiety and discomfort amongst participants and that it would affect large-scale community studies.[89] Some authors reported that they did not have the commonly reported problems of lack of infrastructure or any issues around sound quality, training of personnel and storage/retrieval of recordings.[83] More than three-quarters of ethics committee members from 25 ethics committees felt that the informed consent process was adequate in their institutions, but less than half stated that their ethics committees review AV recordings if there were reports of noncompliance/protocol deviations in the informed consent process.[76]
  - *Role in improving informed consent:* Only few investigators believed that the AV recording of consent process would improve informed consent in one study.[109] However, there was a notion amongst some investigators (and study authors) that the AV recording of informed consent process increases investigator responsibility, accountability and transparency of the process, and that it provides legal protection to participants.[83,89] An observational study that compared informed consent comprehension scores amongst participants before and after the introduction of the mandatory AV recording for the consent process found better comprehension after[78] (the duration between consent and questionnaire administration was shorter in the AV group).

A.6. Bigger picture: Professional (and some lay) participants

Number of studies tagged to topic: 20 (studies and themes that covered cross-cutting ethical issues are included here)

- Seven studies (not included in above sections) that explored larger issues were mostly qualitative studies employing interviews or interviews with focus groups (n=66, 42, 83, 25, 25, 43 participants)[84,90,105,114,115,128] and one mixed methods study (n=80 questionnaires, 14 interviews, 119 informed consent documents).[125]
- Findings from a further 13 studies[67,75,76,90,92,103,108,109,112,127,130,133,140] (methodological aspects included in sections above) that touched upon these larger themes have also been included in this section.

Synthesised findings:

- The seven key studies included here were primarily conducted with professional groups, such as staff from contract research organisations (CROs),[90,115] EC members,[84,125,128] (including judges, social workers, bureaucrats, medico-legal experts),[90] trial sponsors,[90,125] investigators/researchers,[90,125,128] (including academic public health/medical researchers and health

activists from non-governmental/civil society organisations)[114] and employees or participants in research conducted by non-governmental/community organisations.[84] One study included participants from both professional (key informants such as representatives from civil society organisations, community leaders, advocates, services providers, trialists) and lay groups (community members, former trial participants and individuals from HIV high-risk groups).[105] Similarly, of the further 13 studies that have been drawn from, all except two[103,127] were with professional groups and one was with professional and lay participants.[140]

- Compensation (n=10):
    - *Free medicines/vaccines/treatment and post-trial drug access:* A qualitative study reported that lay participants who were educated and from high socio-economic groups felt that the product (vaccine) should be free to motivate participation as it is still being researched and not on sale. However, 'free' meant inferior or dangerous, especially to some from lower socio-economic groups, who compared it to government hospitals being free and providing poor services.[127] There was mixed support for post-trial drug access amongst doctors,[75] investigators[67] and clinical research professionals[133] with many but not all supporting it (in two of these studies, majority of respondents were from industry/private sector and the study authors were from a pharmaceutical company[67] and a contract research organisation[133]).
    - *Payment for participation:* Amongst lay participants with a poorer understanding of research and a higher therapeutic misconception, payment for participation was not acceptable. Some were also sceptical that being paid would mean the sponsor would have lesser responsibility towards them, thereby making the participant more vulnerable. Others felt it was their right or their due, a way of showing appreciation for taking part, an important way to compensate for potential risks/inconvenience, an incentive and a way to make the participant accountable.[127] In another study, investigators supported a reasonable daily/travel allowance for the study visits and emphasised the need to reassure patients that they would not have to pay from their own pockets.[67] In a qualitative study, healthy volunteers were observed bargaining for incentives that were much higher than what was in the protocol and approved by ethics committees.[140]
    - *Payment for researchers:* Amongst lay participants whose motivation for research participation was altruism, there was little support for payment for doctors/researchers to conduct research as they felt that doctors/researchers should also have the same attitude, especially if they were already being paid for their jobs and where the patients' participation was voluntary. Payments for doctors/researchers was felt to be particularly unethical if they were paid per patient recruited. Others felt it was fair for doctors/researchers to be paid for their research work but that this should be reasonable, and were in support of transparency and disclosures regarding payments for doctors/researchers.[127]
    - *Compensation for study-related injuries/serious adverse events:* Most clinical research professionals (sponsors, investigators, ethics committee members) were aware of the Indian laws and guidelines regarding compensation for clinical trial related injuries, but far fewer said they were compliant with them or implemented them.[125] On the other hand, a qualitative study with a similar participant profile reported that key informants (sponsors, investigators, ethics committee members, contract research organisation representatives, programme managers) lacked clarity on the provision of insurance and compensation for trial related injuries and trial participants were completely unaware of compensation arrangement or insurance provisions for trial-related injuries[103] (note: both studies were conducted prior to the introduction of new regulations on compensation in 2013). Most (not all) clinical research professionals (investigators, ethics committee members, sponsors) were in favour of compensation for trial-related injuries/serious adverse events, the new regulations on them and felt able to navigate the stipulated processes, calculations and timelines in relation to these.[76,89,103,133] One study reported that while most clinical research professionals supported compensation for travel, fewer were in support of payments for participants' time, study risks, inconvenience caused by participation or as an incentive for participation.[109] Ethics committee members stated that they did not have the time or the expertise to review compensation plans for trials, although they felt it was important. Also, most were reported as not being aware of the details of insurance contracts, although their review and approval was part of committee members' responsibilities.[125] Some ethics committee members felt that compensation determination should be outside the remit of institutional ethics committees, that defining risk in the compensation formula was challenging and were in support of training for members on the topic.[76] Studies conducted before the new regulations reported that the PI, sponsor and EC members were involved in deciding the level of compensation based on various factors (such as number of dependents, age, type/stage of disease, etc)[103] and that compensation appeared to be limited to acute management of adverse events during the trial (which the patient has to pay for and would be compensated later); clinical research professionals did not mention compensation for lost wages during the adverse event/death and permanent disability, even thought it is mentioned in the national guidelines.[125]

Paramasivan S, *et al. BMJ Global Health* 2021; 6:e004729. doi: 10.1136/bmjgh-2020-004729

- *Adverse event reporting:* A qualitative study reported that key informants lacked clarity on the timelines and process for reporting adverse events. The study reported that in practice it appeared that trial participants were given a list of possible adverse events and numbers to contact if they occurred, but some participants did not report these and sought help from local doctors, which meant they were not reimbursed for their expenses. The authors noted that most adverse events were not recorded as linked to clinical trials and that it appeared that most were recorded primarily for reporting purposes (e.g. to sponsor).[103]

- Sharing of data, blood/tissue samples, results and benefits (n=3): In general, findings acknowledged that there appeared to be limited experience of data sharing and it was perceived as a new territory, amplifying participants' reservations.[84]
  - *Blood/tissue samples sharing:* Lay participants in a qualitative study on clinical trials and biobanking research initially readily agreed to have their blood/tissue samples stored for future research/sharing (as it was 'outside the body' anyway, was a 'waste' for the individual, etc), but were more discerning when probed and given further information.[127] Participants were generally positive about samples being used for future genetic research, but concerns were expressed regarding misuse of samples, ethical issues, commercial exploitations, manipulation of nature and eugenics.[127]
  - *Medical records/clinical data sharing:* This was perceived as non-controversial by lay participants as long as they were used for beneficial purposes and confidentiality was maintained; some however preferred being informed about the reason the records were needed (participants were unaware of legal position on sharing of personal data). While some participants felt that anonymising data would make the sharing of illness/medical history acceptable, others preferred restricted disclosure only to those concerned with research, mainly due to concerns regarding security of electronic information and the stigma around certain health conditions (despite this, participants appeared to prefer being contactable in the case of genetic research, where there was a possibility of individual findings being shared).[127]
  - *What is data?:* The meaning of 'data' was explored in a qualitative study with researchers, managers and research participants (mixed population group comprising professional and lay participants) associated with non-governmental organisations.[84] Data was perceived as including but not limited to demographic/household details, images, videos, medical records and both qualitative and quantitative information. All data was perceived as possibly sensitive as it may have the potential to harm an individual/community/organisation (e.g. HIV status, sexual behaviour), but this mixed population group felt that data could be shared if anonymity could be guaranteed.[84]
  - *Benefits/harms of data sharing:* Benefits of data sharing discussed in the mixed population group included evidence generation, increasing transparency/validity of findings, avoids duplication of efforts and burdening participants with similar research and encouraging learning. Harms of data sharing were mainly the misuse of data, primarily for commercial activities and market research, and the potential for harm to patients/communities, even if data were anonymised, especially when the aims of the data accessor was not clear. It was also felt that participants may refuse to participate in a study or provide incorrect information if they were aware that data may be shared with third parties that they do not know of.[84]
  - *Barriers to data sharing:* Lack of experience, competitive working environments, scepticism of the motives of data accessors and the work required to clean and share data, especially qualitative data were all discussed as barriers to data sharing amongst lay and professional participants.[84] Lay participants indicated that not knowing the individual/institution that would access their data later made it difficult to trust them.[127]
  - *What could help?* Some participants (lay and professional) felt that data sharing would be acceptable when it was with reputed institutions, where it was managed rather than open access to data and with governance/policies in place, including on sharing, authorship, payments, ownership and protection of data from misuse.[84] Lay and professional participants also felt that data sharing was justified if it directly led to interventions or solutions to health issues rather than when it was simply used to write articles (some argued that it would be okay to share data even without direct community benefit if it meant others would learn from it).[84]
  - *Confidentiality:* There was agreement that this was key,[128] with the responsibility for this laying more with the data sharer (i.e. initial researcher) than with the data accessor (i.e. who later requests for access), as the participant trusted the researcher they initially provided consent to.[84] However, this was acknowledged as particularly difficult for qualitative research.
  - *Payment for samples:* Ethics committee members and medical researchers in a qualitative study discussed the ethical dilemmas around paying participants for samples.[128] While making profits out of someone's sample while excluding them from the benefits was not deemed acceptable, being paid was seen as equivalent to tissue trafficking and tissue

being seen as a commercial commodity. Some felt that paying participants could lead to unethical practices, while others felt that it was not acceptable to expect one-sided altruism from participants. They argued that payment for contribution is fair as participants have a right to monetary benefit, especially when the samples led to commercial development and benefit (as opposed to academic research).[128]

- *Benefit sharing:* Lay participants spoke of a community development approach (which involves giving back to the community/medical field, for e.g. through low-cost healthcare) and a participant focussed approach (as the individual agreed to take part when there was uncertainty around the drug) to sharing the commercial profits that were gained by pharma companies after a successful research study.[127] Similar views were expressed by ethics committee members and medical researchers (giving back to the community by supporting further research or healthcare provision in the area, especially when outcomes of studies are commercialised for profits).[128] Giving back to the community as opposed to directly to the individual was also seen as a way of protecting individual confidentiality.[128]
- *Sample ownership:* In the context of biobanking research, sample ownership was seen as a grey area by ethics committee members and medical researchers. It was seen as the patients' (as the needs/interests of the sample contributor were of utmost importance), custodians' (where the storage facility/department/laboratory was the technical owner with responsibility for safe-keeping and prevention of misuse), and the researchers' (as the consent form transfers the ownership from the sample contributor to the researcher). There was also some limited discussion of the difference between ownership of samples/clinical data as opposed to ownership of research data, with former belonging to the patient and latter to the researcher.[128]
- *Disclosing individual findings:* This had not been given much thought of by most ethics committee members and medical researchers, but generally respondents felt that actionable individual results that have clinical significance should be made available to the sample contributor. These views were recognised as being different to countries like the United Kingdom (where individual results were not shared with the contributor), but given the lack of universal health coverage/health insurance and the socio-economic context in India, letting the sample contributor know their results was seen as a way of 'giving back'. It was acknowledged that the mechanisms to carry out this out may be challenging, with suggestions for who could do this ranging from the treating physician, counsellors, social workers or through a special facility that would liaise between the sample contributor and researchers to convey findings and provide counselling (via social workers, not medics).[128] The views of participants regarding disclosing individual findings following biobanking research varied from being unsure (reasons: consent not taken/discussed beforehand, confidentiality violation/sensitive issues, difficulties with insurance), definitely no (reasons: treatment and research are different, findings are irrelevant to patient care, possibility of psychological harm) to definitely yes (reasons: 'giving back', moral obligation, prevention).[128]
- *Consent for data sharing:* Lay participants discussed the need to give participants the option of blanket/general consent or detailed consent at the time of initial consent.[127] While discussing three different types of consent options (namely broad consent, where participants would be told that their data may be shared with others in the future and the research organisation would decide if sharing is appropriate; middle consent, where participants would be told that data may be shared with people from specific research areas; or explicit consent, where participants would be contacted when there was a request for consent), most respondents favoured broad or middle consent. They suggested qualifiers such as informed participants about the possible data accessors.[84]

- <u>Power imbalances (n=17):</u> Unequal power dynamics were explored across different groups and contexts.
  - *Doctor-patient relationship and therapeutic misconception:* Members of the general public did not appear to be familiar with rules and regulations in relation to biomedical research and felt a sense of hopelessness in relation to tackling medical negligence and violations of participant rights due to the differences in power between doctors and patients ('we are small, they are powerful').[127] This power imbalance and a hierarchical paternalistic relationship, along with a doctor's dual role of caregiver-researcher, influence on patient decision making in trial participation and patients' immense trust in a doctor's judgement, especially when they provided assurance about a new unproven treatment (therapeutic misconception), were reported in qualitative studies.[103,127,140] Authors highlighted these as reasons why the informed consent process should be kept away from the treating physician.[103,127] Therapeutic misconception was also reported as more pronounced amongst those from vulnerable groups (e.g. chronically or terminally ill and from lower socio-economic groups), making them more likely to agree to trial participation.[127]

- *Population groups recruited to trials, informed consent and exploitation:* There was a strong view among representatives from civil society organisations and key informants that trial participants were mainly the poor, from rural and tribal communities, who were easy targets as they had limited financial means to access healthcare on their own.[103,114] There was also some suggestion amongst key informants and contract research organisation staff that this was not by chance but a deliberate attempt to recruit from economically disadvantaged groups in slums, targeting mostly unemployed people for volunteer studies as well as, sometimes, Phase III trials.[103,105,127,140] In a qualitative study with healthy volunteers for bioavailability/bioequivalent studies, there appeared to be a unique equation between the volunteers, the middlemen who recruited them and CRO staff. While all the volunteers were from lower socio-economic groups and stated that the financial incentives were their key motivation for research participation (seen as an alternative career prospect), they were aware of the CRO's dependence on them and were observed demanding higher incentives to join or not quit the study, often with the help of the middlemen who recruited them. Volunteers were observed negotiating a better financial deal for their participation, which was much higher than what was in the protocol and approved by ethics committees.[140] However, nearly every ethics committee member and investigator in a qualitative study denied that it was the poor, unemployed, working class and uneducated who were lured into clinical trial participation due to free treatment or other inducements. Some noted that there was no exploitation as many of their poor and illiterate patients were intelligent and asked decisive questions, while others argued that their participants were not rich or poor, but middle class and well aware of their rights.[90] Some staff from contract research organisations insisted that trials that involved such organisations followed the highest standards and that there was no ethical variability in informed consent processes for trials conducted in India as opposed to the West, as any lack of rigour and diligence would not be acceptable to Western sponsors.[90,115] Representatives from civil society organisations, on the other hand, felt that there was ethical variability between trials in the West and in India, framed within the context of fewer ethical guidelines and regulations in India. They also stated that informed consent was majorly compromised and 'meaningless' when the majority cannot access treatment unless they participated in a trial due the failed public health system.[114] Many investigators (mainly recruited from the private sector) agreed that participants agreed to take part in trials to have better access to physicians and/or medical care.[67]
- *West-East, North-South, developed-developing divide:* Frontline health service providers, including some doctors in a qualitative study were reported as feeling that certain types of research (such as HIV vaccines) were concentrated in third world countries as they would not be acceptable in the West.[112] Most clinical research professionals believed that clinical research between developed and developing countries was inequal.[109] Ethics committee members and representatives from civil society organisations viewed Western pharmaceutical trials that recruited from India as a manifestation of the continuing post or neo-colonialist relationship between Western countries and India.[114,121]
- *Are clinical trials relevant to the needs of India?:* Most investigators (from the private sector in a study conducted by authors employed by a pharmaceutical company), felt that studies were relevant to the needs of India and most also believed that the active comparators used in clinical trials in India were usually the same as in the developing world. However, the majority also agreed that pharma companies should set common research goals for all communities and countries.[67] By contrast, ethics committee members believed that pharma companies were using India as a dumping ground to study drugs that are not required for the country's population.[92] Similarly, some investigators in a qualitative study strongly felt that there was a lack of correlation between the disease burden in India and the type of clinical trials that are conducted in the country. Some of them in leadership roles lamented the lack of requests to conduct trials for tropical diseases (although many suffer or die of them) and the large number of trials for conditions that mirror the disease profile in the West (such as diabetes, heart problems, cancer), which is similar to urban India. They therefore felt that trials conducted in India cater to a small segment of the local population and do not benefit the majority of the population, which is poor.[90] This narrative ran counter to the views of executives from contract research organisations who saw clinical trials as benefitting society and their participation in them being about advancing science rather than the pursuit of financial benefits.[90] Some ethics committee members also opined that foreign sponsors should not be expected to take up responsibility for public health in India when the state had itself failed in their social responsibility of delivering healthcare to the majority of the population.[92]
- *Capacity building:* In a qualitative study with employees of contract research organisations, authors noted that in most trials that they studied, the role of these organisations was focused on downstream activities, merely executing the protocols and agendas set by international pharmaceutical companies, following procedures to do the trials 'right' and meticulous documentation (all as part of the phenomenon described by the authors as big-pharmaceuticalisation), with little evidence of locally relevant innovation and knowledge production. However, despite carrying out tasks central to clinical trials, as these organisations delivered a paid service, they had no intellectual property rights and their names did not feature in trial databases or in publications.[115] Representatives of civil society organisations similarly expressed concerns that Indian researchers and

organisations (terms such as 'servants' 'coolies' and 'implementing agency' were used) merely provided labour to produce global data (terms such as 'pre-cooked research' and 'pre-defined research questions' were used) that benefitted the global North and reinforced existing global hierarchies rather than leading on innovation relevant to the local population. Some noted that the Indian researchers doing the research rarely attain leadership roles and when they do, it appears to take a much longer time to break the glass ceiling.[114]

- *Community engagement in research:* Community advocates reported feeling like they were simply being 'used' by research teams to recruit participants to studies without true engagement in all aspects of research. There was also a general mistrust of authorities/researchers conducting or involved in clinical trials, with some questioning why trials needed to be conducted in their countries.[105]
- *Lay participation in ethics committees:* Two-thirds of ethics committee members with a medical background were in favour of including lay people or patients in committee meetings, while only a quarter of members with non-medical backgrounds were in favour of this. Majority of those with non-medical backgrounds stated that their lack of a medical background did not make them feel restricted from participating during committee meetings.[130] However, two-thirds of clinical research professionals felt that lay people were unable to contribute adequately in ethics committee meetings.[133] Similarly, non-medical, non-scientist members of ethics committees in a public hospital expressed difficulties in participating in committee meetings without adequate training and reported feeling like 'show pieces' with an obligatory presence. Medics and scientist members were reported as being the assertive voices due to the hierarchy between medical and non-medical experts and the technical nature of trial protocols. Additionally, some members mentioned that protocols prepared for the technical (or scientific) committees were presented to ethics committees without any adaption or highlighting of ethical issues.[92] Other key reasons mentioned by clinical research professionals for difficulties faced by lay members of ethics committees were lack of training in GCP, regulations and ethical thinking, inadequate exposure/training in clinical research, human rights and compensation, power imbalances (voice can be easily overturned by experts), being unaware of the importance of their role and being used to merely meet quorum requirements.[108,133] Some non-medical experts (social scientists) noted that not being connected to the institution where the research is to be conducted has its advantages as it is easier for non-affiliated members to raise questions than their clinical colleagues who may fear offending their colleagues/institution, but that they have little power to change things.[92]

- <u>Contract research organisations (CROs), civil society organisations (CSOs) and the clinical trial industry (n=7):</u>
  - *Tracing the growth of CROs in India:* One qualitative study that explored the views of CRO staff in relation to a range of ethical issues in clinical trials, outlined the growth of CROs in India.[115] Participants outlined how the pharmaceutical industry, in the pre-TRIPS period, aimed for self-sufficiency as drugs were required in large numbers and clinical trials were not a priority as the focus was on making generics. However, participants observed that more recently, there has been a move towards biosimilars, which involves producing drugs that are similar to, but slightly different or more advanced than, existing drugs. This move from generic drug manufacturing towards innovative research by local pharma companies (which the authors call 'big-pharmaceuticalisation') was seen as a stepping stone towards the development of new chemical entities (although this was perceived as unaffordable to Indian pharmaceutical companies as the industry was not big enough to afford the millions that developing new entities costs). The authors noted that these accounts of progress were embedded within narratives centred on CRO operations/motives and participant safety, with limited mention of the larger ethical issues such as post-trial benefits for participants, compensation or whether the drugs developed provided therapeutic advantages over existing drugs. There was a feeling that the regulatory landscape in India was slow and did not keep up with the fast-paced growth of clinical trials.[115]
  - *CRO operations and collaborative models:* The same qualitative study outlined participants' accounts of the processes by which international pharma companies contact Indian CROs or international CROs with offices in India to conduct trials. CROs advertise their services on various platforms, including online and in conferences, and approach doctors at private and public hospitals and from those listed on the clinical trials registry of India to act as investigators. Participants also discussed six collaborative models between CROs and sponsors and three different types of trials conducted by CROs.[115] Another qualitative study outlined the process through which middlemen were engaged by CROs to recruit healthy volunteers for bioavailability/bioequivalent studies, creating a pool for participants who were regularly approached for participation (serial participation) and often paid more than agreed in protocol/approved by ethics committees in order to retain their ongoing participation.[140]

Paramasivan S, *et al. BMJ Global Health* 2021; 6:e004729. doi: 10.1136/bmjgh-2020-004729

- *Malpractice and scandals:* Most CRO staff were critical of the instances of corruption and malpractices amongst CROs reported in the media, but mainly spoke of these as malpractices by 'others' and never themselves – a narrative that the authors found to be vulnerable as at least one CRO in the study was implicated in a widely reported clinical trial controversy in Bhopal[115] (with evidence of malpractice reported in another study on CROs[140]). Also, while participants were not critical of the new regulations introduced in 2013 following the spate of controversies, they were critical of the lack of government support and protection in the wake of media attacks.[115]
- *Motivations of those involved in clinical trials:* Most CRO executives and investigators stated they were not involved in clinical trials for monetary gains but as a service to science, humanity and society, considering that it involved risks and expressed their unhappiness over the media portrayal of the industry.[90] Some activists expressed concerns about ethics committees becoming financially focussed and providing easy approvals to benefit pharma companies.[114]
- *Views on pharma-sponsored clinical trials:* Nearly two-thirds of doctors believed that trials done for academic purposes, including for dissertation purposes, were relatively more ethical and scientific than industry-sponsored trials and that regulations/legislations related to industry-sponsored trials are inadequate. More than half opined that patients are exploited in industry-sponsored clinical trials.[75] Ethics committee members in a qualitative study were concerned about the role of pharma companies in manipulating clinical trial agreements between the sponsor, investigator and institution, to suit their own interests.[92] By contrast, favourable views regarding pharma-sponsored trials were expressed by investigators (mainly from private sector) in a study authored by researchers in a pharma company. They felt that pharma trials addressed the needs of the community, but agreed that the drugs that were developed were eventually unaffordable to majority of the local population.[67] Representatives from civil society organisations saw commercial, industry-driven clinical trials as having a corrupting effect on many fronts – it lured good investigators away from academic research with the promise of financial benefits and contributed towards good research questions being side-lined if they did not have commercial benefits.[114]
- *India as the preferred destination of choice for clinical trials:* CRO executives and investigators felt that India was preferred not just because it was cost-effective to conduct trials in the country and there was a larger proportion of treatment naïve population, but also because of the high quality of work that was produced by Indian researchers. Others offered more practical reasons such as the need for pharma companies to investigate a drug's pharmacodynamics within non-White population groups before they could be sold to them. Some executives and investigators questioned these narratives and felt that the clinical trial industry was not yet established, that India was not as preferred as was originally predicted and that the population was not as treatment naïve as portrayed due to the common use of over-the-counter medications.[90]
- *Role of CSOs in changing the regulatory landscape in India:* A qualitative study traced the role of health social movements in bringing about more stringent regulations (in 2013) to protect trial participants.[114] Members of CSOs drew from interpretations of social justice and emphasised a rights-based approach to health in their accounts of the activism that brought about key regulatory changes. They acknowledged the importance of randomised controlled trials for the advancement of science, but expressed concerns about the disregard for the wider ethical issues (beyond procedural and informed consent focused agendas) and the perpetuation of existing global hierarchies through pharma companies' choice of drugs, conditions and populations being studied. They stated that pharma companies and CROs are known for their lack of ethical oversight if left to themselves. Some members expressed the challenge in being nuanced or balanced in their debates about clinical trials, while being angry at the injustices in the industry. Some activists spoke about the evolution of their views over time from purely ideological to the more pragmatic, to accommodate a need to move away from dichotomous categorisations based on the funding source for trials (Indian and public being good versus foreign and private being bad).[114]

B. Secondary research
B.1. Secondary research: Primarily documentary

Number of studies tagged to topic: 23

Methodological aspects and limitations:

- Documents studied included informed consent documents (n=138, 30, 50, 300, 119),[64,86,95,107,125] insurance documents (n=18),[125] application forms of research projects submitted to ethics committees (n=100, 73, 100, 445),[85,99,118,120] ethics committee site visit reports (n=7),[119] data/records related to research participants (n=42),[110] ethics approval letters (n=20),[123] other ethics committee governance/administration related documentation (such as approval letters, meeting minutes, project registers/files) where the time period of data collection

was mentioned in place of sample size,[66,96] and data from websites of regulatory, accreditation and registration bodies[106,139,141] (note: one of the articles included here is also in section G).[125]

- Reporting practices in journal articles, journal editorial policies and the clinical trial registry in India are also included here.[63,71,74,93,94,124]

Synthesised findings:

- Completeness, errors and quality of data and documentation in research studies (n=6): The most common issues in research application forms submitted to ethics committees were missing or inadequate information in relation to study titles, participant profile, study benefits, key signatures (investigators, patients), budget details, recruitment methods, compensation for participation or study-related injuries, conflicts of interest, patient safety factors, study documentation, duration of study, sponsoring authority and details on informed consent.[85,118,120] Some of these were reported as more common in academic studies (mainly dissertation projects and some investigator-initiated studies) than sponsored studies (mainly industry sponsored and some government sponsored).[120] One study that examined participant data quality and documentation in investigator-initiated and industry-sponsored studies found that accuracy and data completeness were similar across the two groups, except in documentation related to informed consent processes that were somewhat better in industry-sponsored studies.[110] A study investigating ethics approval letters for compliance with regulations noted the common issues as lack of information on ethics committee members who attended the meeting and their designations, absence of legally required quorum (similar to findings in another study[68]) and legal experts, social scientists or ethicists. Similar to studies above, the issues that were raised by ethics committees in these letters were often in relation to patient recruitment methods as well as other issues such as insurance policies and clinical trial agreements.[123] A study that reported findings from seven site visits[119] observed similar issues to those that reviewed research applications forms submitted to ethics committees.[85,118,120] Authors observed inadequate informed consent documentation (such as missing signatures of patients/PIs and use of forms in local languages that had not been approved by the committee) and delays in reporting of serious adverse events.[119]
- Impact of regulatory changes on registration/accreditation status and composition/structure of ethics committees (n=2): A study of governance/administration related documents in two ethics committees found that the regulatory changes of 2013 had an impact on the structure and functioning of the committees. The number of registered studies reviewed remained the same before and after the regulatory changes, but the number of studies approved decreased. However, there was an increase in turnover time. Similarly, the number of serious adverse events that were reported increased, but the number of meetings to discuss these events increased and the committees' income decreased while their expenses increased. There was also more administrative workload and documentation after the changes.[66] A study that aimed to investigate if the 2013 regulations requiring accreditation and registration (and registration renewal every three years) of ethics committees were adhered to, examined information available on national registration and accreditation databases.[106] The study found that most ethics committees registered were institutional with a fifth being independent, but that the registration numbers may not be reflective of the actual number of committees in India. Of those eligible for re-registration, more of the institutional ethics committee (nearly two-thirds of eligible) were re-registered than the independent ones (less than a third of eligible) and of those that applied for accreditation, less than 10% had received it. The study also found that the distribution of committees across different states was skewed (states with similar populations had large variations in committee numbers – for instance Maharashtra and Bihar with similar populations had more than a quarter and less than 1% of all registered committees respectively). Similar issues arose while comparing registered clinical trials and medical colleges against ethics committees per state, with authors noting that this reflected and perpetuated existing health inequalities across states.[106]
- Reasons for uninitiated studies (n=1): Another study that similarly studied the governance/administrative documents from the same two ethics committees as above found that a greater proportion of pharma-sponsored studies were not initiated after queries raised by ethics committees than investigator-initiated ones. Also, the former had mainly ethical queries raised, while the latter had primarily scientific queries raised by the committees. Most of the ethical issues that were not addressed were related to the informed consent document or processes. Key scientific clarifications required were on sample size, eligibility criteria and inappropriate study design, while ethical queries raised by the committees were in relation to the lack of provision of free investigations or treatments/medicines and patient safety concerns. There appeared to be evidence of 'ethics shopping' as some of the uninitiated studies in multi-centric studies were found to be registered on the trials registry (CTRI) as ongoing or completed at other sites in the country, and these had mainly received ethical queries from the original ethics committees.[96]

- Are clinical trials relevant to the needs of India? (n=2): Two audits of the Clinical Trials Registry of India reported that there was a mismatch between the illnesses researched by clinical trials and the country's disease burden.[139,141] Infectious and parasitic diseases rank first in terms of disease burden but 7th in the number of trials registered in that therapeutic area, while nnon-communicable diseases such as cancer and diabetes mellitus, which rank 6th and 13th in relation to disease burden rank high up in the number of trials registered (ranks 1 and 2 respectively).[139]
- Compensation (n=6):
  - *Compensation for participation:* In a study that aimed to investigate the payments allowed for participation in a trial by ethics committees, authors accessed application forms submitted to committees as well as other relevant documents (study protocols, informed consent documents and correspondence with investigators). They found that nearly all observations studies and a third of interventional studies reviewed by three ethics committees over two years had no mention of compensation for participation. Payments by pharma and government sponsored studies were greater than that by investigator-initiated studies. The most common reasons for payment was reimbursement for travel purposes. Committees had no particular policies or standard operating procedures in place for practices regarding compensation for participation and the amount of compensation approved for participation across studies varied hugely. It also appeared that healthy volunteers were paid more than patients.[99] In another similar study, statements about compensation for participation were not mentioned in nearly all academic studies, more than half the government sponsored studies and in about a third of industry-sponsored studies.[120]
  - *Compensation for study-related injuries/serious adverse events (including their management):* In application forms submitted to two ethics committees over a year, statements related to compensation for injury were not mentioned in nearly all academic and government-sponsored studies and in less than a fifth of industry-sponsored studies.[120] Similarly, a study examining application forms submitted to one ethics committee over more than a year found that statements related to compensation provision if risk occurred was not mentioned in all applications.[85] However, a study investigating informed consent documents submitted to one ethics committee over three years found that information relating to compensation for participants for disability/death from research-related injury (Indian GCP-specified) in informed consent documents was improving over time.[107] A similar observation was made in another study that also examined informed consent documents submitted to three ethics committees over seven years. Authors reported that the documents only mentioned compensation for research-related injuries from 2003 (although the guidelines for this existed from 2000), but that the coverage of the issue in informed consent documents increased from 2003 to 2007.[125] In a similar study examining informed consent documents submitted to two ethics committees over two years, a little over a fifth clearly stated there would be no compensation for trial-related injury, while a little less than half made no mention of it, and some provided caveats, restrictions or ambiguous statements.[64]
  - *Compensation for management of study-related injuries/serious adverse events:* The same study also examined the management of trial-related injuries and found that only a third provided clear statements that free treatment will be provided for trial-related injury, less than a third had no statement on the issue and the rest mentioned restrictions on availing free treatment. Authors also found that the two issues, compensation for trial-related injury and for its management/treatment were sometimes mixed together, making it unclear which aspect was referred to.[64] The ambiguity in the language used to describe compensation for management of study-related injuries in informed consent documents and the variations in the type of compensation offered was also mentioned in another study.[125] Authors also found that compensation for study-related injuries was mainly through 'reimbursement' after proving 'causality' (which was in contrast to national guidelines). They also noted that most insurance documents examined had incomplete details and did not always have their terms and conditions explained.[125]
- Informed consent documents (ICDs) – readability and compliance with legal framework and GCP guidelines (n=4):
  - *Readability:* Two studies evaluated the readability of ICDs used in a clinical research site[86] or those submitted alongside research protocols to ethics committees[107] by employing Western readability tests (Flesch Reading Ease Score and Flesch-Kincaid Grade Level Index). One study employed these tests on Hindi ICDs and reported that the reading level was difficult and that it required graduate level education.[86] The other mentioned English, Hindi and Punjabi ICDs in the article, but it was unclear which ones the tests were applied to, and reported that the readability was close to recommended levels and that there were no changes in readability over three years.[107]

- *Compliance with legal framework:* ICDs from one clinical research site were checked for the presence of the mandatory 19 legally required elements (as per Schedule Y). More than two-thirds of the documents were found to deviate from what the law required. The most common areas that were missing were in relation to appropriate alternative treatments and the voluntary nature of participation. All ICDs mentioned that the study was research, the treatment schedule and random assignment of treatment, risks, measures to protect confidentiality and the tests/procedures that the patient must have within the trial.[95]
- *Compliance with GCP guidelines:* ICDs submitted to one ethics committee over three years (divided into two time periods) were evaluated for compliance with Indian GCP guidelines. Compliance increased over time in relation areas such as basic information (aims, methods), benefits/risks and participant rights. In particular, there was an increase in the mention of contact details of research teams, confidentiality of records, right to withdraw, translation to vernacular languages, and compensation for research-related injuries. There was a decrease in mention of free treatment and alternative treatments over the two time periods.[107]

- Reporting practices (n=6): Studies found that a large number of Indian journal articles did not provide information on ethical approval and/or written informed consent from participants and/or guardians in relation to paediatric, psychiatric and HIV/AIDS research.[63,71,74,94] Other areas that were found to be sub-optimally reported were the obtaining of assent (in paediatric research for children over 7 years old),[63] content and language of consent form and process,[71] financial compensation, non-financial benefits,[94] funding source, conflict of interest[93,124] and dual ethical approval (in the case of research sponsored by a high-income country and conducted in India).[74] One study found that although reporting was sub-optimal, it increased over a period of 7 years (2000 to 2007).[71] Another study that evaluated editorial policies of Indian journals for endorsement of CONSORT statement and ICJME requirements, and the reporting quality of randomised controlled trials in Indian journals in relation to CONSORT statement found these to be less than ideal (although the reporting of ethical issues had improved over the years). Authors also found that methodological and ethical issues were better reported in the clinical trials registry in India than in the journals.[124]
